# The Urinary Metabolome of Healthy Newborns

**DOI:** 10.3390/metabo10040165

**Published:** 2020-04-23

**Authors:** Yamilé López-Hernández, Juan José Oropeza-Valdez, Jorge O. Blanco-Sandate, Ana Sofia Herrera-Van Oostdam, Jiamin Zheng, An Chi Guo, Victoria Lima-Rogel, Rahmatollah Rajabzadeh, Mariana Salgado-Bustamante, Jesus Adrian-Lopez, C. G. Castillo, Emilia Robles Arguelles, Joel Monárrez-Espino, Rupasri Mandal, David S. Wishart

**Affiliations:** 1 CONACyT, Metabolomics and Proteomics Laboratory, Universidad Autónoma de Zacatecas, Zacatecas 98000, Mexico; 2 Unidad de Investigación Biomédica de Zacatecas, Instituto Mexicano del Seguro Social, Zacatecas 98000, Mexico; intrinsection@hotmail.com; 3 CIACYT-Facultad de Medicina, Universidad Autónoma de San Luis Potosí, San Luis Potosí 78210, Mexico; barce_23@hotmail.com (J.O.B.-S.); claudiacastillo@gmail.com (C.G.C.); 4 Biochemistry Department, Faculty of Medicine, Universidad Autónoma de San Luis Potosí, San Luis Potosí 78210, Mexico; chromosomesxx.svo@gmail.com (A.S.H.-V.O.); marianasalgadobustamante@gmail.com (M.S.-B.); 5 The Metabolomics Innovation Center, University of Alberta, Edmonton, AB T6G1C9, Canada; jiamin3@ualberta.ca (J.Z.); aguo@ualberta.ca (A.C.G.); rajabzad@ualberta.ca (R.R.); rmandal@ualberta.ca (R.M.); 6 Hospital Central “Dr. Ignacio Morones Prieto”, San Luis Potosí 78290, Mexico; limamv@hotmail.com; 7 MicroRNAs Laboratory, Unidad Académica de Ciencias Biológicas, Universidad Autónoma de Zacatecas, Zacatecas 98000, Mexico; jalopez@uaz.edu.mx (J.A.-L.); era_arguelles@hotmail.com (E.R.A.); 8 Hospital Christus Muguerza del Parque, Chihuahua 31000, Mexico; jmonarrez@hotmail.com

**Keywords:** newborn, metabolites, tandem mass spectrometry, inborn errors of metabolism, reference values

## Abstract

The knowledge of normal metabolite values for neonates is key to establishing robust cut-off values to diagnose diseases, to predict the occurrence of new diseases, to monitor a neonate’s metabolism, or to assess their general health status. For full term-newborns, many reference biochemical values are available for blood, serum, plasma and cerebrospinal fluid. However, there is a surprising lack of information about normal urine concentration values for a large number of important metabolites in neonates. In the present work, we used targeted tandem mass spectrometry (MS/MS)-based metabolomic assays to identify and quantify 136 metabolites of biomedical interest in the urine from 48 healthy, full-term term neonates, collected in the first 24 h of life. In addition to this experimental study, we performed a literature review (covering the past eight years and over 500 papers) to update the references values in the Human Metabolome Database/Urine Metabolome Database (HMDB/UMDB). Notably, 86 of the experimentally measured urinary metabolites are being reported in neonates/infants for the first time and another 20 metabolites are being reported in human urine for the first time ever. Sex differences were found for 15 metabolites. The literature review allowed us to identify another 78 urinary metabolites with concentration data. As a result, reference concentration values and ranges for 378 neonatal urinary metabolites are now publicly accessible via the HMDB.

## 1. Introduction

According to the 2018 WHO annual report, across the globe, 2.5 million children died in the first month of life. This represents 7000 newborn deaths per day. One third of these deaths occur in the first 24 h of life, while three quarters of infant deaths occur during the first week after birth [1]. Some of the most important causes of neonatal death are preterm birth, intrapartum related events, sepsis/tetanus, infections and congenital abnormalities [2,3]. 

A neonate is formally defined as a baby that is four weeks old or younger. The neonatal period, a time during which a baby is particularly small and fragile, is characterized by continuous and rapid changes in behavior, physiology and metabolism. During the neonatal stage, a number of important physiological events take place, including the establishment of feeding patterns, remodeling of the immune system, changes to the endocrine system, as well as modifications to the infant’s overall metabolism. Usually, the common hospital stay for a newborn is 48 h after a vaginal delivery and 96 h after a caesarean section. However, when medical conditions appear shortly after birth, longer periods of hospitalization are required. It is during these extended stays that multiple hematological, urinary, clinical and biochemical tests are often performed. Therefore, the availability of reference values for hematological, urinary, clinical and biochemical parameters for newborns and neonates is of critical importance. 

Indeed, the use of neonatal reference values for biochemical tests (on blood or urine) of selected metabolites have been the basis of newborn screening programs around the world for more than 50 years. While the first tests, introduced by Dr. Robert Guthrie to diagnose inborn errors of metabolism (IEMs), were quite limited and based on simple enzymatic assays [4], over the past 55 years, these biochemical tests have been expanded and improved significantly. Tandem mass (MS/MS) spectrometry is now widely used in the screening of newborns and has greatly expanded the number of metabolite measurements that can be performed [5]. The specificity and sensitivity of tandem mass spectrometry methods may reach values up to 99.99% and 99%, respectively, for IEM diagnoses. With the advent of LC-MS and direct infusion tandem mass spectrometry (DI-MS/MS), an increased number of less common metabolites and correspondingly rarer IEMs can now be detected with relative ease. 

For full term-newborns, infants and children, a modest number of reference biochemical values are available for blood, serum, plasma, urine and cerebrospinal fluid. This information is also available for preterm newborns [6,7,8,9,10]. However, reference values for many common urinary metabolites in newborns are lacking. Indeed, an in-depth literature review, along with detailed comparisons to values reported in the Human Metabolome Database (HMDB) [11] and the human Urine Metabolome Database (UMDB) [12] indicates that only 212 metabolites have reported urinary reference values for newborns and infants (detected and quantified). In contrast, the number of urinary metabolites with adult reference values is >2000 [12]. This suggests that there is a surprising lack of information about normal urine concentration values for a large number of important metabolites in neonates. 

In the present work, we used targeted MS/MS assays to identify and quantify 136 metabolites of biomedical interest in the urine from 48 healthy, full-term term neonates collected in the first 24 h of life. The targeted assays that we employed use a combination of liquid chromatography tandem mass spectrometry (LC-MS/MS) and flow injection analysis (FIA-MS/MS). In addition to this experimental study, we also performed a detailed literature review (covering the past eight years and a total of 509 papers) to update the information on neonatal urinary concentration data in the HMDB [11] and the UMDB [12]. Combined with the experimental data reported in this study, the total number of neonatal urinary metabolites with reference concentration data has now grown to 378. By conducting this combined experimental/literature review study, we have now nearly doubled the number of urinary metabolites, with reference concentrations for neonates and infants. All of these metabolites and reference values have now been added to the HMDB [11] and the UMDB [12], and are publicly available at www.hmdb.ca. 

## 2. Results

Table 1 shows the clinical and phenotypic data collected from the 48 singleton, healthy full-term neonates, along with the relevant clinical data from the mothers. 

### 2.1. Concentration Data of 136 Metabolites Measured by Targeted Metabolomics

In the present study, we experimentally determined the concentrations of 136 metabolites, selected due to their known involvement in multiple metabolic pathways associated with metabolic or genetic disorders (such as IEMs), that could manifest in the first 24 h of life. A total of 45 amino acids and biogenic amines, 17 organic acids, 24 glycerophospholipids, 10 sphingomyelins and 40 carnitines and acylcarnitines were detected and quantified in every urine sample. Supplementary Table S1 shows the limit of detection (LOD) for each measured metabolite using our LC-MS/MS approach.

**Figure 1:**
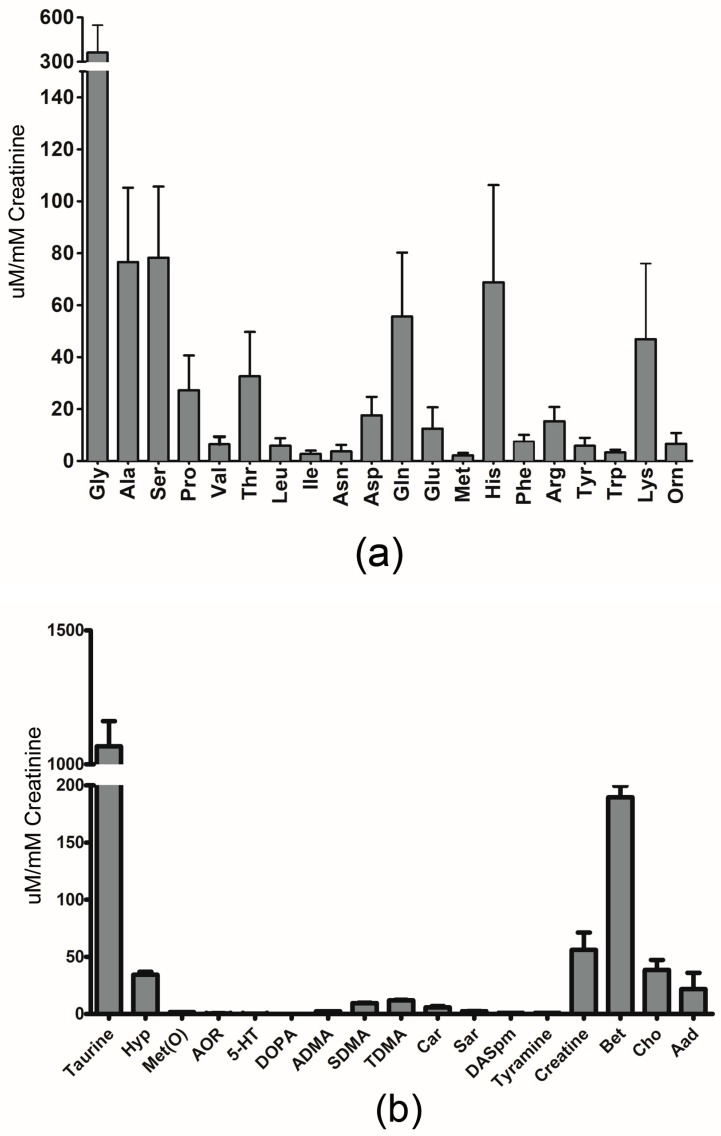

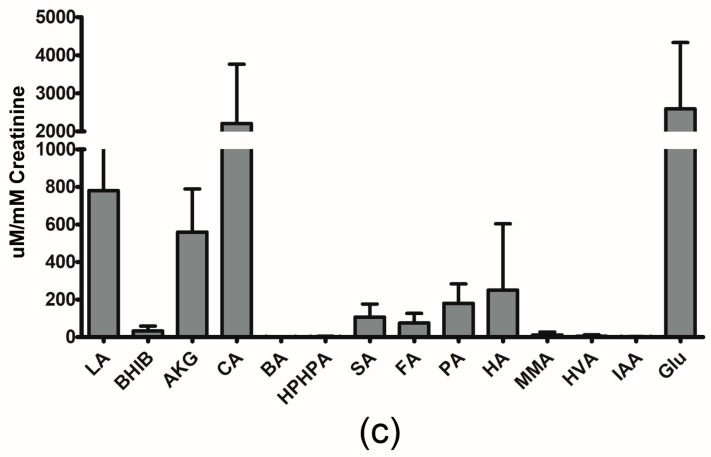
Graphical representation of urinary concentrations of (**a**) amino acids (**b**) biogenic amines and (**c**) organic acids, previously reported in newborns by LC-MS/MS. The error bars reflect one standard deviation. (**a**) Amino acids are represented by their three-letter code. (**b**) 4-hydroxyproline: Hyp; methionine-sulfoxide: Met(O); acetyl-ornithine: AOR; serotonin: 5-HT; asymmetric dimethylarginine: ADMA; symmetric dimethylarginine: SDMA; total dimethylarginine: TDMA; carnosine: Car; sarcosine: Sar; diacethylspermine: DASpm; betaine: Bet; choline: Cho; alpha-aminoadipic acid: Aad. (**c**) lactic acid: LA; 3-Hydroxybutyric acid: BHIB; alpha-Ketoglutaric acid: AKG; citric acid: CA; butyric acid: BA; 3-(3-Hydroxyphenyl)-3-hydroxypropanoic acid: HPHPA; succinic acid: SA; fumaric acid: FA; pyruvic acid: PA; hippuric acid: HA; methylmalonic acid: MMA; homovanillic acid: HVA; indoleacetic acid: IAA; Glucose: Glu.

Figure 1a–c show the quantitative results we obtained using our LC-MS/MS method, expressed as mean ± SD for metabolites previously reported in newborns. Additionally, Supplementary Tables S2 and S3 contain the measured concentration data (mean ± SD, raw and normalized to creatinine) for these metabolites. We also report the urinary reference values found in the literature for these compounds, along with values reported in the HMDB [11] and UMDB [12]. When available, the reference value is listed for newborns or neonates. However, when this data is not available, a reference value listed for infants (which is generally assumed to cover babies aged one week to one year) is provided.

Table 2 and Table 3 contain the experimental results for metabolites not previously reported in newborn and in human urine, respectively, including the absolute concentration (μM, expressed by mean ± standard deviation (SD)); creatinine-normalized values (μM/mM creatinine) expressed as a mean ± SD; and the 2.5–97.5% percentile range (μM/mM creatinine). 

Urinary creatinine (Ucreat) is often used to adjust or normalize urinary analyte concentrations, but we found that Ucreat appeared to be a relatively unreliable reference value in the early newborn period, due to its high variation (ranging from 1000 to 17,000 mM). For this reason, we also provide the absolute concentrations of each metabolite measured in urine.

A total of 86 of these experimentally measured urinary metabolites (64%), along with their concentration ranges, are being reported in neonates/infants for the first time. Of these, 20 metabolites (14% of the compounds identified in this study) are being reported in human urine for the first time ever. Note that the concentration data for some metabolites found through our literature and database searches had more than one reference value, obtained by different laboratories using different methods. This can lead to some minor discrepancies for the numbers reported in these tables. 

### 2.2. Gender-Sex Differences Associated Metabolites

In addition to the combined sex (male + female) results, we also investigated the presence of any sex differences in the measured metabolites. We found that three metabolites (uric acid, butyric acid and octadecadienylcarnitine (C18:2)) were consistently higher (*p* < 0.05) in males than females, while the following 12 metabolites (creatinine, symmetric dimethylarginine, spermine, spermidine, LysoPC a 17:0, LysoPC a 18:1, SM C16:0, SM(OH) C16:1, PC aa 36:0, SM(OH)24:1, PC aa 40:2, and PC aa 40:1) were consistently lower in males than females (*p* < 0.05) (Supplementary Figures S1 and S2). A complete set of tables showing the sex-specific values is included in the Supplementary Material (Supplementary Tables S4–S8). Additionally, the sex-specific values for these urinary metabolites are now available in the HMDB [11] and UMDB [12]. 

### 2.3. Impact of Resolution Mode on the Urinary Metabolome of Healthy Newborns

In the present study, 22 newborns were born by vaginal delivery (VD) and 26 newborns were born by caesarian section (CS). Babies born by VD had significantly lower levels of asparagine, lysine and arginine than babies born by CS. Moreover, an increase in glutaconylcarnitine and nonaylcarnitine was also found in VD babies (Supplementary Figure S3).

### 2.4. Literature Review of the Urinary Metabolome of Healthy Newborns

**Figure 2:**
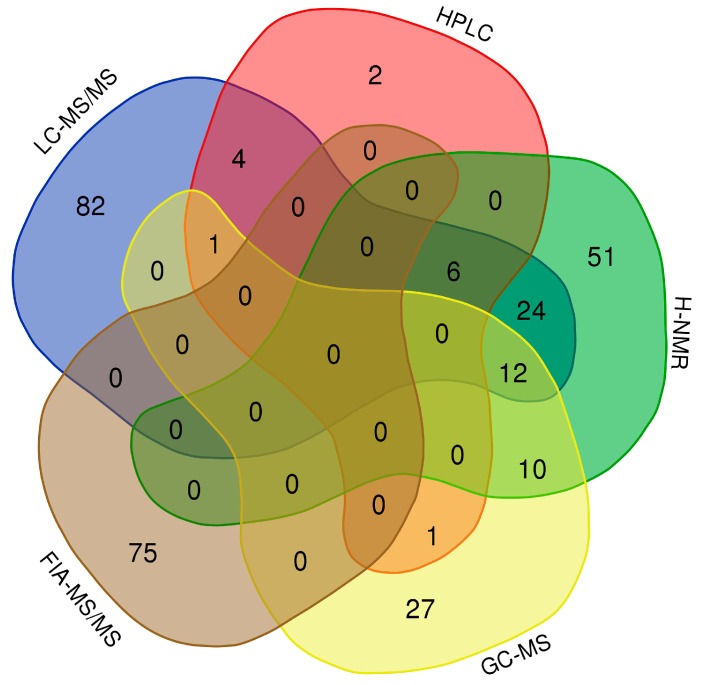
Venn diagram showing the overlap of urine metabolites detected by LC-MS/MS, high performance liquid chromatography (HPLC), nuclear magnetic resonance (^1^H-NMR), gas chromatography mass spectrometry (GC-MS) and FIA-MS/MS. Experimental data: 136 compounds. Literature review: 236 data values from 78 compounds.

Finally, we conducted a thorough literature review of other neonatal/infant metabolite concentrations reported over the past eight years, and used this information to supplement the neonatal urinary data reported in the HMDB and UMDB (www.hmdb.ca). This literature review, which covered an initial set of 509 papers, allowed us to identify another 78 urinary metabolites, with concentration data measured by different platforms (LC-MS/MS, HPLC, ^1^H-NMR, FIA-MS/MS and GC-MS). Combined with the 212 neonatal urinary metabolites previously reported in the HMDB/UMDB and the 86 neonatal urinary metabolites experimentally detected and reported here, there are now 378 neonatal urinary metabolites that have reported concentration values, and which are publicly accessible via the HMDB or UMDB (Figure 2).

## 3. Discussion

Over the past decade, our team, as part of the Human Metabolome Project (HMP), has been systematically characterizing human biofluids using a combination of quantitative metabolomic techniques and literature analyses. During that time, we have characterized human (adult) serum [13], cerebrospinal fluid [14], urine [12], saliva [15] and stool samples [16]. Other groups have also analyzed the metabolomes for human breath [17], breast milk [18], bile fluids [19] and hair [20]. We undertook this study to fill in important gaps in our understanding of urinary metabolite concentrations in neonates. While a number of databases and reference textbooks are available that provide reference values for different populations (age, gender, ethnicity) for serum, plasma, CSF and other fluids in newborns [12,13,21,22,23], there is a paucity of information on urine reference values for newborns and infants. Given that urine samples can be non-invasively collected (via diapers or other simple collection mechanisms) and given that urine provides an invaluable readout of general metabolism, as well as kidney, liver and gut microbiome function, we believe that the development of a reference set of urine metabolites would be highly valuable.

### 3.1. Comparison of Experimental Values with Reported Reference Values 

As described here, we were able to experimentally measure 136 urinary metabolites in 48 full-term healthy neonates. Our combined experimental and literature approach has nearly doubled the total number of metabolites and reference ranges reported for neonatal/infant urine. In addition to greatly expanding the knowledge of neonatal urine composition, we were also able to quantify 20 metabolites (including acylcarnitines and glycerophospholipds), that had never been reported in human urine previously. Given that several well-known metabolic disorders involve the dysregulation of lipid metabolism and molecules transporting lipids, we believe it is very important to monitor the abundance of these types of compounds in biological fluids. In the specific case of the population selected for this study, several babies were born to mothers who, in some cases, were overweight (see Table 1). The monitoring of lipids during the early stages in life may be useful in preventing the development of future metabolic disorders (such as diabetes or dyslipidemia) in the children of overweight or obese mothers. 

In general, the experimental values reported by us are in accordance with previous reference values consulted in the UMDB and the Metagene database (http://metagene.de). We only found notable discrepancies in the concentration values of six compounds: taurine, carnosine, butyric acid, isobutyric acid, 3-hydroxyphenyl-3-hydroxypropionic acid (HPHPA) and indoleacetic acid (Supplementary Table S2). In our study, the interquartile (IQ) range for taurine was 74.64–2841 µmol/mmol creatinine. The reference value reported in the UMDB was 250–910 µmol/mmol creatinine (using NMR) [24]. The abnormal concentration quoted for taurine in the HMDB is 1261 µmol/mmol creatinine, associated with molybdenum cofactor deficiency [25]. However, since all babies included in our study were healthy babies, we must consider other factors contributing to this discrepancy. Relatively few reports on taurine in the perinatal period have been published, but Zaima et al. [26] demonstrated that, in newborns, the urinary taurine concentration was 6222.3 µmol/L on the first day; 1620.1 µmol/L on the third day and on the fifth day 419.3 µmol/L or 1/15 of that of the first day. The daily urinary excretion of taurine was 74.3 micromoles/day on the first day; 79.1 micromoles/day on the third day and 22.7 micromoles/day on the fifth day. So, this suggests that, depending on the day of sampling, the results may be different, with a very large difference between the first and third day after birth. The results obtained in newborns by us correspond to the first 24 h after birth and appear to match well with the data reported by Zaima et al. Taurine accumulates in the maternal tissue during pregnancy to provide the fetus, via the placenta, with adequate levels and to the newborn via breast milk. Low maternal taurine levels result in low fetal levels [27], but high levels of taurine have been found in maternal plasma in the third trimester among GDM Hispanic women treated with insulin, reflecting altered protein metabolism [28].

We also found that the measured urinary concentration of carnosine was consistently lower (IQ 0.47–35.5 µmol/mmol creatinine) than the value reported in previous literature (IQ 3.05–115.4 µmol/mmol creatinine), which was reportedly measured by the same method (LC-MS/MS) [29]. Carnosine is a dipeptide of the amino acids β-alanine and histidine. It is highly concentrated in muscle and brain tissues. It has been reported that carnosine increases with age as the muscle mass increases in newborns [30]. Because the age of the neonates cited in reference [14] is not known, and given that the urinary samples evaluated in the present work belong to the first 24 h of life, we suspect that the concentration differences in carnosine are likely due to minor age differences in the neonate cohorts being sampled, not unlike those seen with taurine. 

In addition to taurine and carnosine, the concentration values of four organic acids (butyric acid, isobutyric acid, 3-hydroxyphenyl-3-hydroxypropionic acid [HPHPA] and indoleacetic acid) are lower than the respective reference values reported for these metabolites (Supplementary Table S2). In the specific case of butyric, isobutyric acid and HPHPA, the literature-derived reference values found by us and listed in Supplementary Table S2 (marked with asterisks) belong to infants (4 weeks to 1 year) and not to neonates (<4 weeks). We suspect that this is the primary reason why our values are significantly lower (by a factor of 5–10) than the reported reference values. It is well known that during the first hours of life, glycogen stores are depleted, and protein catabolism contributes little to energy requirements. This particular metabolic situation seems to be reflected in the reduced urinary excretion of certain organic acids (including butyric and isobutyric acid) compared to older individuals where glycogen stores are fully restored [31]. Furthermore, indoleacetic acid measured in our neonatal cohort were also found to be 10-fold lower than what has been reported in the literature for neonates [32]. Both HPHPA and indoleacetic acid are known gut microbial metabolites, with HPHPA excretion being associated with gut microbial degradation of dietary phenylalanine or polyphenols [33] and indoleacetic acid being a microbially derived breakdown product of tryptophan [33]. Neonates typically do not have a well-established gut microflora, while older infants do. This difference in intestinal microflora likely contributes to the age-related difference in these two microbially-derived metabolites [34].

### 3.2. Gender-Sex Differences Associated Metabolites

Through this study, we were also able to identify a number of metabolites that exhibit clear sex-dependent trends, including uric acid, butyric acid and octadecadienylcarnitine (C18:2), which are increased in males, relative to females. In addition, we found that creatinine, symmetric dimethylarginine, spermine, spermidine, LysoPC a 17:0, LysoPC a 18:1, SM C16:0, SM(OH) C16:1, PC aa 36:0, SM(OH)24:1, PC aa 40:2, and PC aa 40:1 are increased in the urine of females, relative to males. 

The influence of gender in the early newborn metabolome has not been well studied and reliable reference intervals for males and females have not been extensively reported. It is commonly understood that sex differences start in utero [35] and the implementation of sex or gender-dependent strategies in laboratory medicine may help to obtain the correct diagnosis of diseases affecting newborns. Only a handful of studies have been published addressing the effect of gender in the urinary newborn metabolome [30,36,37]. In particular, Diaz et al. [37] reported that allantoin and xanthine levels are higher in the urine of females than in males, which suggests a slightly altered nitrogen metabolism in females, compared to males. In addition, glucose and lactose were also found to be higher in females, but infant females also had lower levels of inositols and other sugars, suggesting that changes in sugar metabolism may be associated with gender in the first days of life [37]. More recently, Caterino et al. [36] analyzed urinary organic acids in healthy Caucasian infants and children (aged 1 month to 36 months), and reported that in the first six months of life, sex differences were more frequent, and the majority of urinary organic acids were higher in males than in females. The authors conclude that sex deeply influences urinary organic acids levels [36]. In our study, we only found significant sex differences in the level of two organic acids: uric acid and butyric acid (higher in males than in females). 

### 3.3. Impact of Birth Resolution on the Urinary Metabolome of Healthy Newborns

We found significantly lower levels of asparagine, lysine and arginine in the urine of neonates born by VD, than in those born by CS. Moreover, an increase in glutaconylcarnitine and nonaylcarnitine was found in the urine of VD neonates. Early in the postnatal period, major physiological adaptations occur in newborns to cope with stress and extrauterine cold exposure upon exiting the womb. Differences found in relation to delivery mode have been associated with difference in lower gut microflora colonization as well as in alterations of hepatic metabolism [37]. In a recent study, Pierre Martin et al. [38] reported that VD newborns had lower urinary levels of lysine (as we found), as well as lower levels of histidine, relative to CS newborns. It is also important to remember that lysine is involved in carnitine biosynthesis. Indeed, the two main precursors for carnitine biosynthesis are lysine and methionine, which provide the carbon backbone and 4-N-methyl groups of carnitine, respectively. The substrate for carnitine biosynthesis is 6-N-trimethyllysine (TML) [39]. Using up reserves of lysine to produce TML to synthesize carnitine and acylcarnitines would be expected to lead to low levels of lysine. This is consistent with the increased urinary excretion of acylcarnitines and the decreased excretion of lysine in VD newborns found by us. 

It is also possible that these metabolic differences may arise from the differential nutrition between VD and CS newborns. Neonates from our cohort born by VD were fed with breast milk immediately after delivery. Breast milk is known to be rich in carnitine. The development of ketogenesis in the human neonate is greatly dependent on the exogenous supply of carnitine, because the liver has a limited capacity for de novo carnitine synthesis [40]. Given that VD versus CS metabolic differences were not the primary focus of this study, it is clear that additional mechanistic studies are warranted.

### 3.4. Urinary Metabolites Associated with IEMs

While most IEM tests are designed for blood or dried blood spots, many health institutions also perform urine tests using targeted assays that employ liquid or gas chromatography coupled with tandem mass spectrometry [41,42]. In a recent study, Kennedy et al. [43] identified (but did not quantify) over 1200 molecules from among 100 clinical urine samples from children (average age of 4.3 years). This study showed clear biochemical signatures for 16 of the 18 IEM diseases tested. This work nicely illustrated the utility of urinary metabolomics for assessing IEMs [43]. Supplementary Table S9 lists the metabolites included in our experimental approach that have been previously identified as urinary markers for different IEMs.

A number of IEMs, such as those related to amino acid metabolism; creatinine disorders; fatty acid metabolism and β-oxidation and organic acid disorders; peroxisomal biogenesis and metabolism; aminolevulinic acid dehydratase deficiency; purine and pyrimidine metabolism and urea cycle disorders, can be diagnosed via urinalysis. Urinary metabolic profiling can be used to detect altered levels of intermediate metabolites that result from the incomplete metabolism of amino acids or organic acids. 

Carnitines and acylcarnitines are usually measured in plasma to detect IEMs. However, genetic defects in the OCTN2 carnitine transporter can result in a condition known as primary carnitine deficiency. This is associated with a decreased accumulation of intracellular carnitine, higher levels of carnitine in the urine and low levels of carnitine in serum. Because carnitine is transferred from the mother to the child via the placenta, shortly after birth, the levels of carnitine in newborns with carnitine transporter defects could, artefactually, be normal. However, this deficiency could be more easily diagnosed in urine, by detecting an increase in urinary carnitine. Therefore, to properly detect this condition, the additional analysis of urinary organic acids in conjunction with the clinical presentation would allow one to correctly diagnose it [44]. In the study reported by Kennedy et al. [43], they measured urinary concentrations of β-hydroxyisovaleroylcarnitine, α-hydroxyisovalerylcarnitine, tiglylcarnitine, succinylcarnitine, malonylcarnitine, 3-methylglutarylcarnitine and glutarylcarnitine. These authors demonstrated the comparable usefulness of these acylcarnitine biomarkers when determined both in plasma and in urine, for the correct diagnosis of holocarboxylase deficiency and lysinuric protein intolerance [43]. However, to use urinary acylcarnitines in the diagnosis of IEMs, it is necessary to have reference values for normal concentrations of urinary acylcarnitines. Prior to our work (presented in Table S6), reference concentration values for most neonatal urinary acylcarnitines were not previously available. 

In this work, we also provide the urinary concentration values for 24 glycerophospholipids and 10 sphingomyelins. There are at least 40 IEMs with neurological/muscular presentations related to the defects in phospholipid, sphingolipid and long chain fatty acid biosynthesis [45]. Additionally, there are more than 100 IEMs that may lead to primary or secondary defects of complex lipids synthesis and remodeling [46]. Supplementary Table S9 shows the associated IEMs to urinary markers experimentally measured in the present work and the abnormal concentrations reported in literature to diagnose these conditions. We believe that, with the availability of so much more referential data for neonatal urine, it should now be possible to identify a number of other previously undetected or unsuspected IEMs or metabolic conditions in neonates via urinalysis. 

### 3.5. The Importance of Age-Specific Intervals

The availability of quantitatively measured metabolites in biofluids of newborns, particularly in urine, provides extensive and dynamic information that, if followed and controlled over time, can be useful in predicting the “biological age” of infants. Biological age (as opposed to calendar age) is greatly influenced by several factors, such as diet, stress, environment, lifestyle, genetics and disease [47]. Since these factors play important roles in determining the metabolome, urinary metabolomics may provide reliable and sensitive markers to understand the complexity of age-related changes, leading to the identification of novel treatments or strategies for the management of health and disease in early childhood [48]. These age-specific data may also be used for the establishment of inflection points related to metabolic disorders, which is very important in accurate disease diagnosis or prognosis. In this sense, it is important to keep in mind that metabolite concentrations change continuously with growth and age, especially with children and infants. This is often not fully realized, due to the fact that the majority of clinical studies that have been published are for adults [49,50,51,52,53,54,55]. Indeed, to date, there have only been a few comprehensive studies looking at age-specific intervals in children [48,56,57] and newborns [30]. 

Among the urinary metabolites that are known to vary considerably with age are carnitine, 3-hydroxyisovalerate, creatinine, alanine, and trigonelline. Indeed, these metabolites differ significantly between younger and older groups [54]. Urinary trimethylamine-*N*-oxide (TMAO) has been found to be higher in infants (aged 1 week–1 year), which may be directly associated to the consumption of milk at this age and/or the corresponding gut microflora found in infants, due to breast feeding [48].

Urinary glycine and glutamine levels have been reported to decline significantly within the first year of life, probably due to their use in supporting the increased growth of skeletal muscle tissue during infancy [48]. Another important metabolite measured in urine that changes with age is creatinine (Ucreat). In particular, it has been noted that creatinine levels increase as children age. However, only a few studies have attempted to measure the values of Ucreat in the first days of life. It has been demonstrated that the mean Ucreat concentration is significantly higher in neonates than in older children [58]. Furthermore, Ucreat is also highly variable, until it begins to stabilize by the first month. Creatinine levels at birth typically do not yet reflect neonatal creatinine clearance, but rather maternal creatinine clearance. Furthermore, because of passive tubular back leak in infants instead of active secretion, creatinine clearance does not yet fully reflect GFR (glomerular filtration rate).

Supplementary Table S10 shows a comparison between metabolite concentrations (normalized to creatinine) measured in the present study and in a previous study conducted by our group on healthy adult urine samples [12]. By limiting the comparison to samples analyzed in the same lab using largely the same techniques and workflow (from pre-analytical to post-analytical analysis), we could ensure that any inter-laboratory variation was minimized. From a total of 79 common metabolites, 33 showed a clear, decreasing trend with age, while most others were largely unchanged. In comparing adult urine with newborn urine, we found that a number of amino acids were much higher in newborns than adults, including glycine, alanine, serine, proline, histidine, lysine, methionine, and most branched-chain amino acids. Amino acids have been previously reported to decrease with age and this phenomenon likely arises from age-dependent changes in cell growth, tissue growth and muscle metabolism [59]. High levels of glycine, serine and proline in newborn urine may be correlated with generally high levels of these amino acids in the newborn body, as high levels of water-soluble metabolites in the urine strongly correlated with levels of water-soluble metabolites in the blood or tissues. These small amino acids are likely needed to support high levels of collagen synthesis in newborns (the most abundant protein in the body). High levels of essential amino acids in newborn urine also reflect their high levels throughout the newborn body. Essential amino acids are obviously needed by newborns to support rapid cell division and cell growth during the first weeks of life [60]. Another group of molecules that showed a decreasing trend with age are biogenic amines. This is in line with previous reports. For example, in children (at five years of age), muscle carnosine levels are initially low but, as children grow, carnosine levels gradually increase before declining and reaching a plateau in adulthood [61]. Dopamine is also observed to be lower in adults than in newborns. This decrease may be explained by the age-related decline in the integrity of the dopamine system, which is seen in most adults [62]. Urinary polyamines, such as putrescine, spermidine and spermine, were also seen to decline with age in our newborn-adult comparison. This result mirrors a previous study that showed that polyamines declined progressively with age [63]. Polyamines are typically elevated in cells that are rapidly dividing (as might be expected for newborns) and can often be seen as metabolic by-products in cancer (in adults). 

## 4. Materials and Methods 

### 4.1. Sample Collection and Research Ethics Approvals

This was a cross-sectional study carried out at the Hospital Central “Dr. Ignacio Morones Prieto”, San Luis Potosi, Mexico, from January 2018 to August 2019. The study was approved by the Research and Ethics Committee, with the registration number 84-17 and folio CONBIOETICA-24-CEI-001-201604279. The protocol complied with the Declaration of Helsinki. Written informed consent was obtained from the parents of all studied subjects. 

At the moment of birth, all newborns were carefully examined by a trained neonatologist. Variables such as weight, sex, Apgar score at 1 min, Apgar score at 5 min, pregnancy resolution, gestational age (Capurro test) and Silverman–Anderson test scores were determined and recorded for each newborn as expediently as possible. After this assessment was complete, one urine sample was collected noninvasively for each newborn. The genitals were cleaned thoroughly, and a sterile bag was placed on the genital area until micturition. 

Urine samples contaminated with meconium were discarded. After visual inspection of the urine sample and after at least 1 mL of urine had been excreted by the infant, the sterile bag was removed. The urine sample in the bag was transferred via a micropipette to a sterile 1.5 mL Eppendorf tube. The urine samples were then centrifuged at 3000 rpm to precipitate sediments. Following this, the samples were stored in sterile microtubes at −80 °C until further use.

### 4.2. Chemicals and Internal Standards (ISTD)

Optima™ LC/MS grade formic acid and HPLC grade water were purchased from Fisher Scientific (Ottawa, ON, Canada). Optima™ LC/MS grade ammonium acetate, phenylisothiocyanate (PITC), 3-nitrophenylhydrazine (3-NPH), HPLC grade methanol and HPLC grade acetonitrile (ACN) were also purchased from Sigma-Aldrich (Oakville, ON, Canada). Furthermore, ^2^H-, ^13^C-, and ^15^N-labelled compounds were purchased from Cambridge Isotope Laboratories, Inc. (Tewksbury, MA, USA) and from Sigma-Aldrich (Oakville, ON, Canada).

A working internal standard (ISTD) solution mixture in water (for amino acids, biogenic amines, carbohydrates, carnitines and derivatives, phosphatidylcholines and their derivatives) was made by mixing all the prepared isotope-labeled stock solutions together. For organic acids, a working internal standard (ISTD) solution mixture in 75% aqueous methanol was made. All standard solutions were aliquoted and stored at −80 °C until further use.

### 4.3. Metabolite Measurement

#### 4.3.1. Sample Preparation

The urine samples were thawed on ice before analysis. For the analysis of organic acids, 10 μL of an internal standard (ISTD) mixture solution and 10 μL of the samples (three phosphate buffered saline [PBS] blank samples, seven calibration standards, three quality control samples and urine samples) were pipetted directly into the center of corresponding spot in a 96-deep well plate. 30 μL of 75% aqueous methanol was then added to each of the wells, followed by adding 25 μL to each of the following three solutions: 1) 3-nitrophenylhydrazine (250 mM in 50% aqueous methanol), 1-Ethyl-3-(3-dimethylaminopropyl)-carbodiimide (150 mM in methanol) and pyridine (7.5% in 75% aqueous methanol). The whole plate was then shaken at room temperature for 2 h to derivatize the organic acids. After the derivatization reaction, to each plate well, 350 μL of water and 25 μL of butylated hydroxytoluene solution (2 mg/mL in methanol) were added, to dilute and stabilize the final solution. Then, 10 μL was injected into an Agilent 1260 UHPLC-equipped QTRAP 4000 mass spectrometer for LC-MS/MS analysis, using multiple reaction monitoring (MRM) scanning in the negative mode. For the analysis of amino acids, biogenic amines and derivatives, acylcarnitines, lipids and glucose, 10 μL of each sample and 10 μL of the ISTD mixture solution were loaded onto the center of a 96-well filter plate and dried in a stream of nitrogen for 30 min. Subsequently, 50 μL of 5% phenylisothiocyanate (PITC) solution was added to each sample and the whole plate was then incubated at room temperature for 20 min. After incubation, all the samples were again dried under nitrogen for 1.5 h to evaporate excess PITC. The extraction of targeted metabolites was then achieved by adding 300 μL of extraction solvent (5 mM ammonium acetate prepared in methanol). The extracts were obtained by centrifugation into a lower 96-deep well collection plate. To quantify amino acids, biogenic amines and derivatives, extracts were diluted with water in a 1:1 ratio, and 10 μL was injected; to analyze acylcarnitines, lipids and glucose, extracts were diluted five times and 20 μL was injected into an Agilent 1260 UHPLC-equipped QTRAP 4000 mass spectrometer, using multiple reaction monitoring (MRM) scanning in the positive mode. All data analysis was done using Analyst 1.6.2 (AB SCIEX, Foster, CA, USA) and MultiQuant 3.0.3 (AB SCIEX, Foster, CA, USA).

#### 4.3.2. LC-MS/MS Method

An Agilent reversed-phase Zorbax Eclipse XDB C18 column (3.0 mm × 100 mm, 3.5 μm particle size, 80 Å pore size), with a Phenomenex (Torrance, CA, USA) SecurityGuard C18 pre-column (4.0 mm × 3.0 mm), was used for the LC-MS/MS analysis of organic acids, amino acids, biogenic amines and derivatives. 

The LC parameters used for the analysis of amino acids, biogenic amines and their derivatives were as follows: mobile phase A 0.2% (*v*/*v*) formic acid in water, and mobile phase B 0.2% (*v*/*v*) formic acid in acetonitrile. The gradient profile was as follows: *t* = 0 min, 0% B; *t* = 0.5 min, 0% B; *t* = 5.5 min, 95% B; *t* = 6.5 min, 95% B; *t* = 7.0 min, 0% B; and *t* = 9.5 min, 0% B. The column oven was set at 50°C. The flow rate was 500 μL/min, and the sample injection volume was 10 μL.

For the analysis of organic acids, the mobile phases used were A) 0.01% (*v*/*v*) formic acid in water, and B) 0.01% (*v*/*v*) formic acid in methanol. The gradient profile was as follows: *t* = 0 min, 30% B; *t* = 2.0 min, 50% B; *t* = 12.5 min, 95% B; *t* = 12.51 min, 100% B; *t* = 13.5 min, 100% B; *t* = 13.6 min, 30% B and finally maintained at 30% B for 4.4 min. The column oven was set to 40 °C. The flow rate was 300 μL/min, and the sample injection volume was 10 μL.

#### 4.3.3. FIA-MS/MS Method

For the analysis of lipids, acylcarnitines and glucose, the LC autosampler was connected directly to the MS ion source by red PEEK tubing. The mobile phase was prepared by mixing 60 μL of formic acid, 10 mL of water and 290 mL of methanol; and the flow rate was programmed as follows: *t* = 0 min, 30 μL/min; *t* = 1.6 min, 30 μL/min; *t* = 2.4 min; 200 μL/min; *t* = 2.8 min, 200 μL/min; and *t* = 3.0 min, 30 μL/min. The sample injection volume was 20 μL.

#### 4.3.4. Quantification

To quantify organic acids, amino acids, biogenic amines and derivatives, an individual seven-point calibration curve was generated for each analyte. The ratios of each analyte’s signal intensity to its corresponding isotope-labelled internal standard were plotted against the specific known concentrations, using quadratic regression with a 1/x^2^ weighting.

Lipids, acylcarnitines and glucose were analyzed semi-quantitatively. Single point calibration of a representative analyte was built, using the same group of compounds that share the same core structure, assuming linear regression through zero. All data analysis was done using Analyst 1.6.2 and MultiQuant 3.0.3.

### 4.4. Statistical Analysis

GraphPad Prism 5.0 software (GraphPad Software, Inc., La Jolla, CA, USA) was used for all statistical analyses reported here. Percentiles, mean values and standard deviations (SD) were calculated using standard statistical formulas. Continuous and categorical variables are presented as mean ± SD or median (interquartile range) and number (for percentile) respectively. The Kolmogorov–Smirnov test was used to test the normality of the distribution for continuous variables. A statistical analysis to evaluate gender differences was performed with GraphPad Prism 5.0 software (GraphPad, La Jolla, CA, USA) and MetaboAnalyst (https://www.metaboanalyst.ca). *p*-value adjustments for multiple metabolites were carried out by using Benjamini-Hochberg false discovery rate adjustment (FDR < 0.05). 

The methodology for determining the reference intervals is based on the recommendations from the International Federation of Clinical Chemistry [64]. All primary reference ranges have been calculated using more than 40 samples, allowing for reliable estimates of the 2.5th and 97.5th centiles. A non-parametric calculation was used to calculate ranges, due to non-normally distributed data.

### 4.5. Literature Review and Data Entry

The last detailed literature overview conducted by our team on urinary metabolites in newborns was performed in 2012 [12]. A literature search was conducted in PubMed from November 2019 to February 2020. Our first search filter combined the truncated search term “urin*”. Our second search filter combined the term “newborn” and its synonym “neonat*” with the Boolean operator OR. The third search filter combined the terms “reference value” and “concentration” with the operator OR. Our last search filter used the truncated search term “metaboli*”. We then combined these searches with the Boolean operator AND. Through this preliminary search, we retrieved 509 articles. From this number, 191 were from the last 8 years (the time of the last literature update conducted on the UMDB and HMDB for neonatal reference values). After careful reading through these articles, 34 papers were found to have useful information about metabolite concentration values in newborn urine. From these 34 papers, 78 metabolites with concentration values were obtained and used to update the HMDB/UMDB.

## 5. Conclusions

The size and chemical diversity of the “measurable” metabolome of healthy neonates appear to be somewhat smaller and simpler than that of children or adults (~300 metabolites vs. ~2000 metabolites). This appears to be due to fundamental differences in neonatal metabolism, as well as differences due to diet, exposures and gut microflora composition. Despite the relative simplicity of neonatal urine in terms of its metabolic diversity, we were surprised by the appearance of several unexpected metabolites. Indeed, 86 of the experimentally measured urinary metabolites had not previously been reported in neonates/infants, and another 20 metabolites are being reported in human urine for the first time ever. Comparisons between neonatal urine and adult urine also show some striking concentration differences for certain metabolites. Much higher levels of essential amino acids, collagen-associated amino acids and acylcarnitines in neonatal urine likely reflect the large pool of these compounds needed to sustain rapid cell growth and cell division in neonates. In addition to the obvious differences between adult and infant urine composition, we also observed clear differences in urinary metabolites between newborn males and females, as well as differences arising due to birth modality (VD vs. CS). Some of our findings reiterated the findings of earlier studies, while others appear to be quite novel. The clear existence of sex differences in the urine composition of newborns reinforces the need for implementing specific sex-reference values of metabolic markers for female and male neonates. These sex-specific differences may also be relevant in the diagnosis of IEMs. Interestingly, prior to this study, the influence of sex on acylcarnitines, glycerophospholipids, biogenic amines and sphingomyelines urinary levels at birth had not been investigated.

One of the major strengths of our work is the fact that all the urinary samples were collected in the same period of time (the first 24 h of life), which makes the reported measurements more comparable and homogeneous. As a result, we believe that the values reported here should constitute a robust and generally useful set of clinical reference values for the absolute and creatinine-normalized urinary concentration for healthy, full-term neonates. All of these values, along with chemical structures and detailed descriptions of the compounds, are freely available in the UMDB and HMDB (www.hmdb.ca). We hope that such a set of reference values and reference information will be used by clinicians and other health professionals to assist with the diagnosis, prognosis or monitoring of various IEMs and other neonatal health conditions.

## Figures and Tables

**Table 1:** Clinical data from the newborns and their mothers.

**Newborns (*N* = 48)**	
Sex	
Female	17 (35%)
Male	31 (65%)
Gestational age (weeks)	38.2 ± 1.5
Ave. Apgar at 1 min	8 (100%)
Ave. Apgar at 5 min	9 (100%)
Ave. Silverman–Anderson	0 (100%)
Weight (g)	2979 ± 472
Weight (g) males	2992 ± 86.59
Weight (g) females	2882 ± 112.4
**Mothers (*N* = 48)**	
BMI (pre-gestational)	27.1 ± 4.9
Age (years)	29 ± 7
Resolution	
Vaginal delivery	22 (45.8%)
Caesarean section	26 (54.2%)
Euglycemic	22 (45.8%)
Gestational Diabetes Mellitus (GDM)	26 (54.2%)

**Table 2:** Metabolites not previously reported in newborns.

Metabolite	HMDB ID	Mean ± SD (μM)	Mean ± SD (μM/mM Creatinine)	2.5–97.5% Percentile (μM/mM Creatinine)
Histamine	HMDB0000870	0.08 ± 0.03	0.02 ± 0.01	0.01–0.04
Putrescine	HMDB0001414	1.09 ± 1.54	0.31 ± 0.63	0.03–3.42
Methionine sulfoxide	HMDB0002005	7.02 ± 4.70	1.60 ± 0.73	0.60–3.60
N2-Acetylornithine	HMDB0003357	3.28 ± 4.00	0.67 ± 0.75	0.12–3.50
Serotonin	HMDB0000259	0.98 ± 0.67	0.20 ± 0.06	0.10–0.35
DOPA	HMDB0000181	0.20 ± 0.10	0.05 ± 0.03	0.01–0.15
Asymmetric dimethylarginine	HMDB0001539	10.6 ± 6.40	2.31 ± 0.83	0.98–4.90
Symmetric dimethylarginine	HMDB0003334	45.7 ± 30.8	9.80 ± 3.00	5.33–18.1
Spermidine	HMDB0001257	0.25 ± 0.19	0.06 ± 0.05	0.02–0.30
Spermine	HMDB0001256	0.23 ± 0.28	0.06 ± 0.11	0.01–0.62
Diacetylspermine	HMDB0002172	4.64 ± 3.52	1.03 ± 0.59	0.37–3.11
Trimethylamine *N*-oxide	HMDB0000925	59.4 ± 54.0	12.2 ± 10.3	0.30–43.1
*p*-Hydroxyhippuric acid	HMDB0013678	37.1 ± 24.8	8.03 ± 3.48	4.20–19.6
LysoPC a C16:1	HMDB0010383	0.013 ± 0.011	0.003 ± 0.003	0.0001–0.0150
LysoPC a C16:0	HMDB0010382	0.19 ± 0.20	0.043 ± 0.036	0.007–0.169
LysoPC a C17:0	HMDB0012108	0.018 ± 0.013	0.005 ± 0.001	0.0008–0.0582
LysoPC a C18:2	HMDB0010386	0.03 ± 0.04	0.007 ± 0.007	0.001–0.030
LysoPC a C18:0	HMDB0010384	0.06 ± 0.06	0.015 ± 0.012	0.0008–0.0652
LysoPC a C20:4	HMDB0010395	0.02 ± 0.04	0.005 ± 0.006	0.0–0.03
PC ae C36:0	HMDB0013406	0.02 ± 0.03	0.006 ± 0.013	0.0–0.08
PC aa C36:6	HMDB0008206	0.007 ± 0.006	0.002 ± 0.003	0.0–0.02
PC aa C36:0	HMDB0007886	0.05 ± 0.06	0.013 ± 0.023	0.001–0.130
PC aa C38:6	HMDB0008116	0.08 ± 0.12	0.017 ± 0.020	0.002–0.089
PC aa C38:0	HMDB0007893	0.08 ± 0.04	0.022 ± 0.021	0.005–0.124
PC ae C40:6	HMDB0013422	0.02 ± 0.02	0.005 ± 0.004	0.001–0.021
PC aa C40:6	HMDB0008057	0.04 ± 0.05	0.010 ± 0.013	0.0–0.07
SM(OH) C14:1	HMDB0013462	10.1 ± 0.09	0.02 ± 0.04	0.00–0.25
SM C16:1	HMDB0013464	0.10 ± 0.11	0.02 ± 0.02	0.0–0.11
SM C16:0	HMDB0010168	2.02 ± 2.28	0.50 ± 0.92	0.07–5.31
SM(OH) C16:1	HMDB0013463	0.06 ± 0.08	0.02 ± 0.04	0.0–0.24
SM C18:1	HMDB0012101	0.08 ± 0.10	0.02 ± 0.02	0.0–0.06
SM C18:0	HMDB0012087	0.31 ± 0.37	0.07 ± 0.79	0.01–0.43
SM C20:2	HMDB0013465	0.005 ± 0.005	0.001 ± 0.001	0.0–0.004
SM(OH) C22:2	HMDB0013467	0.03 ± 0.04	0.01 ± 0.01	0.0–0.05
SM(OH) C22:1	HMDB0013466	0.16 ± 0.15	0.04 ± 0.03	0.0–0.15
SM(OH) C24:1	HMDB0013469	0.04 ± 0.05	0.01 ± 0.02	0.0–0.14
Carnitine (C0)	HMDB0000062	8.96 ± 7.07	2.01 ± 1.13	0.79–5.67
l-Acetylcarnitine (C2)	HMDB0000201	4.49 ± 4.32	0.89 ± 0.38	0.38–1.86
Propenoylcarnitine (C3:1)	HMDB0013124	0.08 ± 0.08	0.02 ± 0.01	0.0–0.06
Propionylcarnitine (C3)	HMDB0000824	0.14 ± 0.09	0.03 ± 0.02	0.01–0.07
Butenylcarnitine (C4:1)	HMDB0013126	0.09 ± 0.04	0.02 ± 0.01	0.01–0.04
Butyrylcarnitine (C4)	HMDB0002013	0.53 ± 0.39	0.12 ± 0.07	0.05–0.33
Hydroxypropionyl carnitine (C3OH)	HMDB0013125	0.11 ± 0.05	0.03 ± 0.01	0.01–0.06
Tiglylcarnitine (C5:1)	HMDB0002366	0.34 ± 0.23	0.07 ± 0.03	0.03–0.16
Hydroxybutyryl carnitine (C4OH)	HMDB0002095	0.18 ± 0.10	0.04 ± 0.02	0.01–0.08
Hexenoylcarnitine (C6:1)	HMDB0013161	0.06 ± 0.03	0.020 ± 0.004	0.01–0.02
Hexanoylcarnitine (C6)	HMDB0000756	0.12 ± 0.07	0.03 ± 0.08	0.02–0.05
Hydroxyvalerylcarnitine (C5OH)	HMDB0013132	0.36 ± 0.20	0.08 ± 0.05	0.04–0.29
Glutaconylcarnitine (C5:1DC)	HMDB0013129	0.07 ± 0.04	0.02 ± 0.01	0.0–0.03
Glutarylcarnitine (C5DC)	HMDB0013130	0.20 ± 0.11	0.05 ± 0.02	0.02–0.09
Octanoylcarnitine (C8)	HMDB0000791	0.24 ± 0.19	0.05 ± 0.05	0.02–0.31
Methylglutarylcarnitine (C5MDC)	HMDB0000552	0.27 ± 0.16	0.06 ± 0.02	0.03–0.13
Nonaylcarnitine (C9)	HMDB0013288	0.46 ± 0.38	0.09 ± 0.04	0.03–0.18
Pimelylcarnitine (C7DC)	HMDB0013328	0.21 ± 0.14	0.05 ± 0.02	0.02–0.09
Decenoylcarnitine (C10:1)	HMDB0013205	0.34 ± 0.12	0.09 ± 0.04	0.03–0.20
Decanoylcarnitine (C10)	HMDB0000651	0.29 ± 0.18	0.06 ± 0.02	0.04–0.13
Dodecenoylcarnitine (C12:1)	HMDB0013326	0.24 ± 0.10	0.06 ± 0.02	0.02–0.14
Dodecanoylcarnitine (C12)	HMDB0002250	0.35 ± 0.27	0.08 ± 0.04	0.03–0.25
Tetradecadienyl carnitine (C14:2)	HMDB0013331	0.05 ± 0.03	0.010 ± 0.003	0.01–0.02
Tetradecenoylcarnitine (C14:1)	HMDB0013329	0.06 ± 0.03	0.01 ± 0.02	0.0–0.1
Tetradecanoylcarnitine (C14)	HMDB0005066	0.11 ± 0.10	0.02 ± 0.02	0.01–0.10
Hydroxytetradecadienylcarnitine (C14:2OH)	HMDB0013332	0.03 ± 0.02	0.007 ± 0.002	0.003–0.014
Hydroxytetradecenoyl carnitine (C14:1OH)	HMDB0013330	0.04 ± 0.02	0.008 ± 0.002	0.004–0.015
Hexadecadienyl carnitine (C16:2)	HMDB0013334	0.02 ± 0.01	0.004 ± 0.002	0.0–0.01
Hexadecanoylcarnitine (C16)	HMDB0000222	0.05 ± 0.03	0.01 ± 0.01	0.01–0.06
Octadecadienylcarnitine (C18:2)	HMDB0006469	0.010 ± 0.003	0.003 ± 0.001	0.0–0.01

**Table 3:** Metabolites not previously reported in human urine.

Metabolite	HMDB ID	Mean ± SD (μM)	Mean ± SD (μM/mM Creatinine)	2.5–97.5% Percentile (μM/mM Creatinine)
LysoPC a C14:0	HMDB0010379	0.02 ± 0.02	0.006 ± 0.003	0.002–0.012
LysoPC a C18:1	HMDB0002815	0.07 ± 0.09	0.02 ± 0.03	0.0–0.20
LysoPC a C20:3	HMDB0010394	0.014 ± 0.002	0.004 ± 0.003	0.0–0.01
LysoPC a C24:0	HMDB0010405	0.07 ± 0.02	0.02 ± 0.01	0.0–0.05
LysoPC a C26:1	HMDB0029220	0.01 ± 0.01	0.002 ± 0.002	0.0–0.01
LysoPC a C26:0	HMDB0029205	0.01 ± 0.01	0.004 ± 0.005	0.0–0.03
LysoPC a C28:1	HMDB0029221	0.01 ± 0.01	0.002 ± 0.002	0.0–0.01
LysoPC a C28:0	HMDB0029206	0.04 ± 0.01	0.011 ± 0.007	0.0–0.04
PC aa C32:2	HMDB0007874	0.03 ± 0.03	0.01 ± 0.01	0.0–0.03
PC aa C40:2	HMDB0008276	0.01 ± 0.01	0.003 ± 0.008	0.0–0.05
PC aa C40:1	HMDB0007993	0.03 ± 0.04	0.01 ± 0.02	0.0–0.13
Decadienylcarnitine (C10:2)	HMDB0013325	0.14 ± 0.07	0.03 ± 0.01	0.01–0.07
Dodecanedioylcarnitine (C12DC)	HMDB0013327	0.27 ± 0.29	0.05 ± 0.05	0.01–0.29
Hexadecenoylcarnitine (C16:1)	HMDB0006317	0.05 ± 0.02	0.01 ± 0.01	0.0–0.03
Hydroxyhexadecadienylcarnitine (C16:2OH)	HMDB0013335	0.02 ± 0.01	0.004 ± 0.001	0.0–0.01
Hydroxyhexadecenoyl carnitine (C16:1OH)	HMDB0013333	0.04 ± 0.02	0.010 ± 0.004	0.0–0.02
Hydroxyhexadecanoylcarnitine (C16OH)	HMDB0061642	0.04 ± 0.02	0.008 ± 0.003	0.0–0.02
Octadecenoylcarnitine (C18:1)	HMDB0006464	0.02 ± 0.01	0.005 ± 0.004	0.0–0.03
Octadecanoylcarnitine (C18)	HMDB0000848	0.03 ± 0.03	0.01 ± 0.01	0.0–0.07
Hydroxyoctadecenoylcarnitine (C18:1OH)	HMDB0013339	0.02 ± 0.01	0.004 ± 0.002	0.0–0.01

## Supplementary Materials

The following are available online at https://www.mdpi.com/2218-1989/10/4/165/s1, Figure S1: Metabolites showing higher urinary concentration levels (*p* < 0.05) in female versus male. Data are shown as Box and Whisker plots. Comparison of continuous variables were performed using the t-test (when data were normally distributed) or Mann-Whitney test (when data were non-normally distributed), Figure S2: Metabolites showing higher urinary concentration levels (*p* < 0.05) in male versus female. Data are shown as Box and Whisker plots. Comparison of continuous variables were performed using the *t*-test (when data were normally distributed) or Mann-Whitney test (when data were non-normally distributed), Figure S3: Metabolites differentially expressed in vaginal delivery (VD) and Caesarean section (CS) newborns. Data are shown as Box and Whisker plots. Comparison of continuous variables were performed using the *t*-test (when data were normally distributed) or Mann-Whitney test (when data were non-normally distributed); Table S1: Limit of detection for measured analytes, Table S2: Metabolites previously reported in newborns and infants, Table S3: Creatinine urinary concentration in newborns. Table S4: Differences in gender (Amino acids and biogenic amines); Table S5: Differences in gender (Organic acids and glucose); Table S6: Differences in gender (Glycerophospholipids); Table S7: Differences in gender (Sphingomyelins); Table S8: Differences in gender (acylcarnitines); Table S9: List of metabolites experimentally measured in the present work that are currently used as primary or secondary markers for Inborn errors of metabolism, Table S10: Comparison between newborn and adult urinary concentration of 79 metabolites measured via LC-MS/MS.
