# Supplementary material for: The Urinary Metabolome of Healthy Newborns
Source: Metabolites. 2020 Apr 23;10(4):165. doi: 10.3390/metabo10040165 (PMC7240964; doi:10.3390/metabo10040165)
Supplement: Supplementary file 1 [file metabolites-10-00165-s001.zip › sp/SUPPLEMENTARY FIGURE 1.pdf]

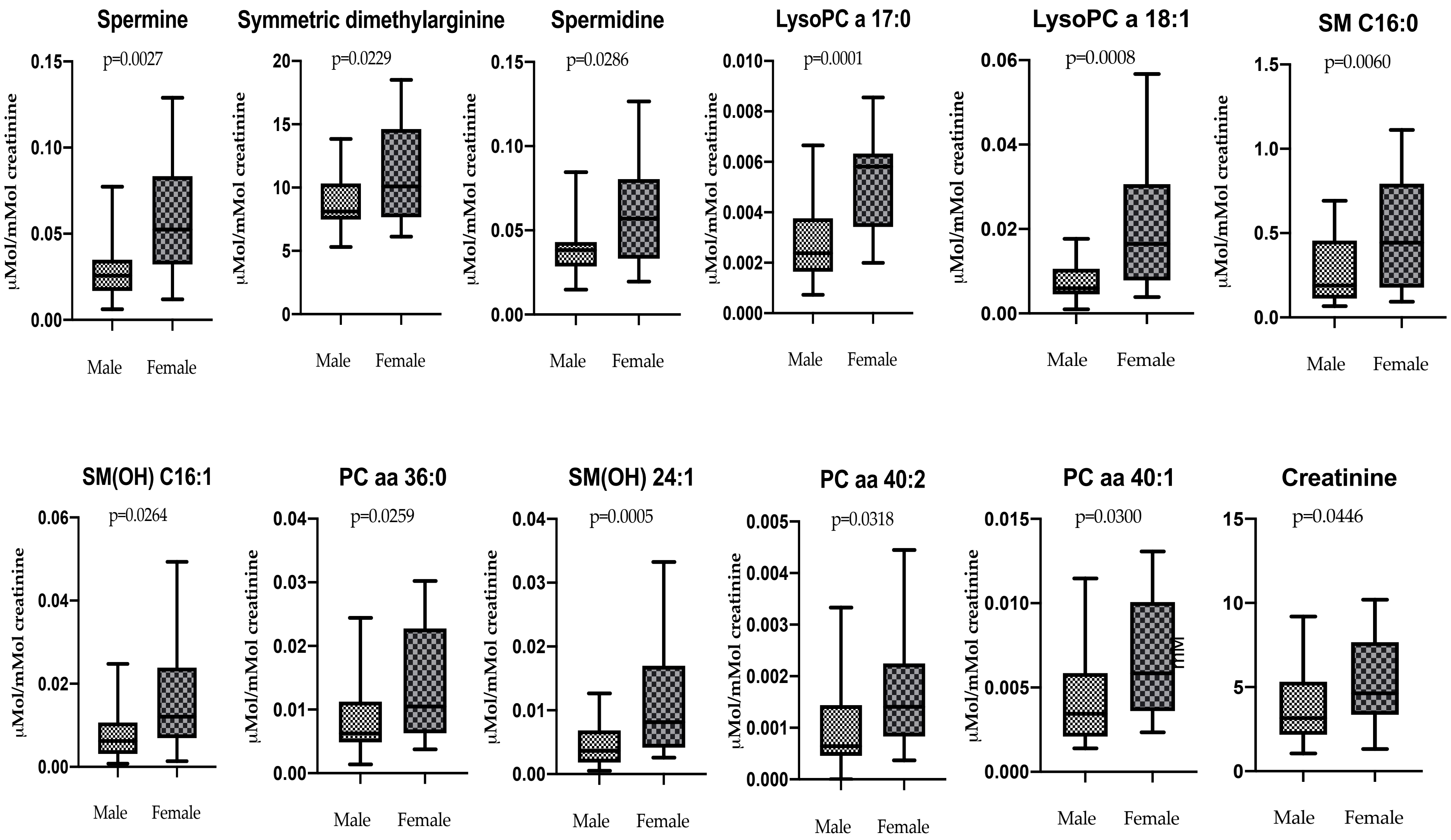

Supplementary Figure 1. Metabolites showing higher urinary concentration levels ($p < 0.05$) in female versus male. Data are shown as Box and Whisker plots. Comparison of continuous variables were performed using the t-test (when data were normally distributed) or Mann-Whitney test (when data were non-normally distributed).
