# Supplementary material for: The Urinary Metabolome of Healthy Newborns
Source: Metabolites. 2020 Apr 23;10(4):165. doi: 10.3390/metabo10040165 (PMC7240964; doi:10.3390/metabo10040165)
Supplement: Supplementary file 1 [file metabolites-10-00165-s001.zip › sp/Supplementary Table 1. Limit of detection for measured analytes.pdf]

Supplementary Table 1. Limit of detection for measured analytes

**Analyte**	**LOD (μM)**
Creatinine	6.34
Glycine	0.97
Alanine	0.55
Serine	0.307
Histamine	0.00698
Proline	0.0945
Valine	0.205
Threonine	0.556
Taurine	0.611
Putrescine	0.0625
4-Hydroxyproline	0.0857
Leucine	0.789
Isoleucine	0.306
Asparagine	0.472
Aspartic acid	2.73
Glutamine	0.422
Glutamic acid	0.967
Methionine	0.191
Dopamine	0.0109
Histidine	0.142
a-Aminoadipic acid	0.735
Phenylalanine	0.0671
Methionine sulfoxide	0.268
Arginine	0.0318
N2-Acetylornithine	0.0647
Serotonin	0.0164
Tyrosine	0.0818
DOPA	0.00674
Asymmetric dimethylarginine	0.0169
Symmetric dimethylarginine	0.0139
Tryptophan	0.0470
Kynurenine	0.0286
Carnosine	0.0362
Ornithine	0.0439

Lysine	0.131
Spermidine	0.0125
Spermine	0.0103
Sarcosine	0.143
N1,N12-Diacetylspermine	0.0128
Tyramine	0.0371
Creatine	0.198
Betaine	0.701
Choline	1.33
Trimethylamine N-oxide	0.631
1-Methylhistidine/3-Methylhistidine	0.0840
Lactic acid	1.04
3-Hydroxybutyric acid	0.205
alpha-Ketoglutaric acid	0.258
Citric acid	2.58
Butyric acid	0.133
HPHPA	0.0495
p-Hydroxyhippuric acid	0.269
Succinic acid	0.121
Fumaric acid	0.0135
Pyruvic acid	0.0628
Isobutyric acid	0.0832
Hippuric acid	0.493
Methylmalonic acid	0.0309
Homovanillic acid	0.393
Indoleacetic acid	0.852
Uric acid	1.37
Glucose	8.37
LysoPC a C14:0	1.25
LysoPC a C16:1	0.124
LysoPC a C16:0	0.235
LysoPC a C17:0	0.115
LysoPC a C18:2	0.241
LysoPC a C18:1	0.107
LysoPC a C18:0	0.149
LysoPC a C20:4	0.176

LysoPC a C20:3	0.143
LysoPC a C24:0	0.422
LysoPC a C26:1	0.0922
LysoPC a C26:0	0.105
LysoPC a C28:1	0.0519
LysoPC a C28:0	0.349
SM(OH) C14:1	0.119
SM C16:1	0.0211
SM C16:0	0.0396
SM(OH) C16:1	0.0128
SM C18:1	0.0171
PC aa C32:2	0.0847
SM C18:0	0.0361
SM C20:2	0.203
PC ae C36:0	0.0379
PC aa C36:6	0.0348
PC aa C36:0	0.068
SM(OH) C22:2	0.0279
SM(OH) C22:1	0.0268
PC aa C38:6	0.0164
PC aa C38:0	0.677
PC ae C40:6	0.101
SM(OH) C24:1	0.0291
PC aa C40:6	0.0974
PC aa C40:2	0.0125
PC aa C40:1	0.299
Carnitine (C0)	4.24
L-Acetylcarnitine (C2)	0.602
Propenoylcarnitine (C3:1)	0.101
Propionylcarnitine (C3)	0.0853
Butenylcarnitine (C4:1)	0.0419
Butyrylcarnitine (C4)	0.108
Hydroxypropionylcarnitine (C3OH)	0.0387
Tiglylcarnitine (C5:1)	0.0463
Valerylcarnitine (C5)	0.0549
Hydroxybutyrylcarnitine (C4OH)	0.0404

Hexenoylcarnitine (C6:1)	0.0552
Hexanoylcarnitine (C6)	0.127
Hydroxyvalerylcarnitine (C5OH)	0.0397
Glutaconylcarnitine (C5:1DC)	0.0302
Glutarylcarnitine (C5DC)	0.0206
Octanoylcarnitine (C8)	0.206
Methylglutarylcarnitine (C5MDC)	0.0312
Nonaylcarnitine (C9)	0.0225
Pimelylcarnitine (C7DC)	0.0208
Decadienylcarnitine (C10:2)	0.0171
Decenoylcarnitine (C10:1)	0.127
Decanoylcarnitine (C10)	0.0382
Dodecenoylcarnitine (C12:1)	0.0644
Dodecanoylcarnitine (C12)	0.0218
Tetradecadienylcarnitine (C14:2)	0.0113
Tetradecenoylcarnitine (C14:1)	0.0689
Tetradecanoylcarnitine (C14)	0.0724
Dodecanedioylcarnitine (C12DC)	0.0877
Hydroxytetradecadienylcarnitine (C14:2OH)	0.0789
Hydroxytetradecenoylcarnitine (C14:1OH)	0.0927
Hexadecadienylcarnitine (C16:2)	0.0239
Hexadecenoylcarnitine (C16:1)	0.0384
Hexadecanoylcarnitine (C16)	0.0117
Hydroxyhexadecadienylcarnitine (C16:2OH)	0.0654
Hydroxyhexadecenoylcarnitine (C16:1OH)	0.0119
Hydroxyhexadecanoylcarnitine (C16OH)	0.0799
Octadecadienylcarnitine (C18:2)	0.0954
Octadecenoylcarnitine (C18:1)	0.0899
Octadecanoylcarnitine (C18)	0.0676
Hydroxyoctadecenoylcarnitine (C18:1OH)	0.0658
