# Supplementary material for: The Urinary Metabolome of Healthy Newborns
Source: Metabolites. 2020 Apr 23;10(4):165. doi: 10.3390/metabo10040165 (PMC7240964; doi:10.3390/metabo10040165)
Supplement: Supplementary file 1 [file metabolites-10-00165-s001.zip › sp/supplementary Table 10 comparisson urine adult newborn-DSW.pdf]

Supplementary Table 10. Comparison between newborn and adult urinary concentration of 79 metabolites measured via LC-MS/MS

Metabolite	Newborn urine (μM/mM creatinine)	Adult urine (μM/mM creatinine)
Creatinine*	4,952.0± 3,381.0	12,475 ± 7,955
Glycine	102.6-853.6	101.0 (37.0–250.6)
L-Alanine	32.2-147.8	21.7 (7.2–47.4)
L-Arginine	6.82-30.5	7.4 (3.2–14.6)
L-Asparagine	0.42-10.5	8.8 (4.6–17.7)
L-Aspartic acid	5.57-34.6	10.9 (1.9–26.8)
L-Glutamic acid	1.96-35.3	6.4 (0.6–17.5)
L-Glutamine	16.2-134.4	39.9 (18.4–72.5)
L-Histidine	21.1-188.1	45.6 (21.7–71.5)
L-Isoleucine	0.29-7.30	1.3 (0.5–2.7)
L-Leucine	0.33-13.4	3.0 (1.6–6.0)
L-Lysine	10.5-140.5	17.9 (3.6–56.1)
L-Methionine	0.54-4.81	0.8 (0.4–1.6)
L-Ornithine	1.44-24.4	4.4 (1.2–22.1)
L-Phenylalanine	4.14-14.2	5.9 (2.8–10.8)
L-Proline	9.5-78.2	1.1 (0.1–2.5)
L-Serine	32.2-145.5	24.5 (12.3–50.2)
L-Threonine	9.24-96.2	14.6 (6.6–29.3)
L-Tryptophan	1.90-6.03	5.6 (9.3–2.1)
L-Tyrosine	2.10-14.8	8.7 (3.0–20.1)
L-Valine	1.0-18.4	4.3 (2.4–10.4)
Acetyl-ornithine	0.12-3.50	1.3 (0.5–2.8)
Alpha-aminoadipic acid	1.23-15.5	7.2 (2.5–16.4)
Asymmetric dimethylarginine	0.98-4.90	2.7 (1.4–4.2)
Carnosine	0.47-35.5	1.2 (0.2–4.1)
Dopamine	0.13-2.63	0.4 (0.2–0.7)
Histamine	0.01-0.04	0.03 (0.01–0.10)
Kynurenine	0.02-0.71	0.41 (0.08–1.31)
L-Dopa	0.01-0.15	0.02 (0.01–0.04)
Methioninesulfoxide	0.60-3.60	0.31 (0.14–0.66)
Putrescine	0.03-3.42	0.20 (0.04–0.40)
Sarcosine	0.55-9.98	1.6 (0.2–9.4)
Serotonin	0.10-0.35	0.08 (0.04–0.12)
Symmetric dimethylarginine	5.33-18.1	2.8 (2.0–9.4)
LysoPC a C16:0	0.007-0.169	0.014**
LysoPC a C17:0	0.0008-0.0582	0.0032 (0.0008–0.0087)
LysoPC a C18:0	0.0008-0.0652	0.0040 (0.0013–0.0100)
LysoPC a C20:4	0.0-0.03	0.0020 (0.0005–0.0038)
PC aa C36:6	0.0-0.02	0.0037**
PC aa C38:6	0.002-0.089	0.0010 (0.0005–0.0036)
SM(OH) C14:1	0.0-0.25	0.0028 (0.0008–0.0049)
SM C16:1	0.0-0.11	0.0009 (0.0004–0.0023)
SM C16:0	0.07-5.31	0.0100 (0.0016–0.0700)
SM(OH) C16:1	0.0-0.24	0.0006 (0.0001–0.0023)
SM C18:1	0.0-0.06	0.0004 (0.0001–0.0013)
SM C18:0	0.01-0.43	0.0024 (0.0011–0.0070)
SM C20:2	0.0-0.004	0.0003 (0.0–0.0016)
SM(OH) C22:2	0.0-0.05	0.0007 (0.0001–0.0028)
SM(OH) C22:1	0.0-0.15	0.0023 (0.0009–0.0058)
SM(OH) C24:1	0.0-0.14	0.0010 (0.00034–0.0025)
Carnitine (C0)	0.79-5.67	4.5 (0.6–15.2)
L-Acetylcarnitine (C2)	0.38-1.86	2.0 (0.4–5.9)

Propenoylcarnitine (C3:1)	0.0-0.06	0.003 (0.001–0.006)
Propionylcarnitine (C3)	0.01-0.07	0.07 (0.01–0.20)
Butenylcarnitine (C4:1)	0.01-0.04	0.014 (0.008 –0.021)
Butyrylcarnitine (C4)	0.05-0.33	0.76 (0.12–1.81)
Hydroxypropionylcarnitine (C3OH)	0.01-0.06	0.008 (0.004–0.012)
Tiglylcarnitine (C5:1)	0.03-0.16	0.10 (0.05–0.20)
Valerylcarnitine (C5)	0.02-0.29	0.220 (0.037–0.440)
Hexenoylcarnitine (C6:1)	0.01-0.02	0.019 (0.009–0.050)
Hexanoylcarnitine (C6)	0.02-0.05	0.05 (0.03–0.07)
Glutaconylcarnitine (C5:1DC)	0.0-0.03	0.04 (0.01–0.06)
Glutarylcarnitine (C5DC)	0.02-0.09	0.04 (0.03–0.10)
Octanoylcarnitine (C8)	0.02-0.31	0.05 (0.03–0.09)
Methylglutarylcarnitine (C5MDC)	0.03-0.13	0.030 (0.016–0.052)
Nonaylcarnitine (C9)	0.03-0.18	0.20 (0.07–0.46)
Pimelylcarnitine (C7DC)	0.02-0.09	0.03 (0.01–0.11)
Decenoylcarnitine (C10:1)	0.03-0.20	0.14 (0.06–0.32)
Decanoylcarnitine (C10)	0.04-0.13	0.04 (0.02–0.06)
Dodecenoylcarnitine (C12:1)	0.02-0.14	0.028 (0.011–0.042)
Dodecanoylcarnitine (C12)	0.03-0.25	0.02 (0.01–0.04)
Tetradecadienylcarnitine (C14:2)	0.01-0.02	0.0023 (0.0010–0.0050)
Tetradecenoylcarnitine (C14:1)	0.0-0.1	0.003 (0.001–0.006)
Tetradecanoylcarnitine (C14)	0.01-0.10	0.018 (0.001–0.088)
Hydroxytetradecadienylcarnitine (C14:2OH)	0.003-0.014	0.003 (0.001–0.008)
Hydroxytetradecenoylcarnitine (C14:1OH)	0.004-0.015	0.0020 (0.0008–0.0040)
Hexadecadienylcarnitine (C16:2)	0.0-0.01	0.0010 (0.0005–0.0040
Hexadecanoylcarnitine (C16)	0.01-0.06	0.0022 (0.0020–0.0030)
Octadecadienylcarnitine (C18:2)	0.0-0.01	0.0007 (0.0003–0.0020)

*Concentration of metabolite (Mean±SD) is expressed by μM

**no reliable concentration
