# Supplementary material for: The Urinary Metabolome of Healthy Newborns
Source: Metabolites. 2020 Apr 23;10(4):165. doi: 10.3390/metabo10040165 (PMC7240964; doi:10.3390/metabo10040165)
Supplement: Supplementary file 1 [file metabolites-10-00165-s001.zip › sp/Supplementary Table 2 and 3. Metabolites previously reported in newborns and infants.pdf]

Supplementary Table 2. Metabolites previously reported in newborns and infants

Metabolite	HMDB ID	Mean ± SD µM	Mean ± SD (µM/mM creatinine)	2.5% - 97.5% Percentile (µM/mM creatinine)	Literature values (µM/mM creatinine)
Glycine	HMDB0000123	1581±957.3	362.1±185.5	102.6-853.6	113-1427[a] [1]
Alanine	HMDB0000161	353.5±261.1	76.6±28.6	32.2-147.8	10.5-340.1[a] [2]
Serine	HMDB0000187	354.9±207.8	78.3±27.4	32.2-145.5	180 (25.3-334.7)[c] [3]
Proline	HMDB0000162	126.9±86.4	28.5±15.4	9.5-78.2	8-260[a] [1]
Valine	HMDB0000883	31.2±25.6	6.75±3.40	1.0-18.4	1.2-23.9[a] [2]
Threonine	HMDB0000167	151.4±110.4	34.0±19.5	9.24-96.2	2.83-137.6[a] [2]
Taurine	HMDB0000251	5526±5919	1067±649.5	74.6-2841	250-910[a] [4]
4-Hydroxyproline	HMDB0000725	136.1±63.6	34.3±18.3	8.12-92.2	4-230[a] [1]
Leucine	HMDB0000687	27.9±24.1	5.95±2.87	0.33-13.4	6.67 (0-13.33)[c] [3]
Isoleucine	HMDB0000172	13.3±11.1	2.86±1.44	0.29-7.30	3.75 ± 2.75[b] [5]
Asparagine	HMDB0000168	14.07±9.35	3.72±2.5	0.42-10.5	0.0-5.0[a] [6]
Aspartic acid	HMDB0000191	78.9±47.7	17.6±7.2	5.57-34.6	25.1 (20-30.2)[c] [3]
Glutamine	HMDB0000641	269.5±203.4	57.2±26.6	16.2-134.4	15.7-337.6[a][2]
Glutamic acid	HMDB0000148	61±69.6	12.5±8.21	1.96-35.3	37.8 (8.89-66.7)[c] [3]
Methionine	HMDB0000696	11.0±9.3	2.24±0.89	0.54-4.81	4.45 (0-8.89)[c] [3]
Histidine	HMDB0000177	373.4±364.6	71.4±41.2	21.1-188.1	16.4-433.5[a] [2]
a-Aminoadipic acid	HMDB0000510	33.3±27.9	6.95±3.91	1.23-15.5	1.1-31.1[a] [2]
Phenylalanine	HMDB0000159	37.8±32.1	7.58±2.50	4.14-14.2	14.7 (5.33-24)[c] [3]
Arginine	HMDB0000517	73.5±54.2	15.3±5.47	6.82-30.5	1.1-63.3[a][2]
Tyrosine	HMDB0000158	26.9±18.7	5.91±3.05	2.10-14.8	4.41-77.5[a][2]
Tryptophan	HMDB0000929	15.7±10.5	3.36±0.98	1.90-6.03	1.6-35.6[a] [2]
Carnosine	HMDB0000033	31.1±49.7	5.81±7.60	0.47-35.5	3.05-115.4[a][2]
Ornithine	HMDB0000214	29.3±20.4	7.07±4.93	1.44-24.4	0-29.9[a] [2]
Lysine	HMDB0000182	190.2±103.3	46.9±29.1	10.5-140.5	3-170[a] [1]
Sarcosine	HMDB0000271	10.93±9.57	2.43±1.72	0.55-9.98	0-8.48[a][2]
Tyramine	HMDB0000306	4.32±2.67	0.95±0.49	0.30-2.54	0.0[a] [4]
Creatine	HMDB0000064	209.2±325.1	56.0±105.1	4.66-455.3	25-776[a] [7]
Betaine	HMDB0000043	780.5±311.8	189.6±69.8	74.1-410.3	44-1200[a] [4]
Choline	HMDB0000097	92.39±103.2	18.32±11.05	3.99-50.7	0-94[a] [8]
1-Methylhistidine<br>3-Methylhistidine	HMDB0000001<br>HMDB0000479	299.4±287.8	54.9±28.4	18.6-145.2	1.92-47.4[a]<br>9.95-39.6[a] [2]
Lactic acid	HMDB0000190	741.5±779.5	166.9±171.8	47-774.2	0-540[a] [9]
3-Hydroxybutyric acid	HMDB0000357	27.8±29.5	5.52±5.15	0.72-23.3	2.0 (0.5-9.8)[c] [10]
alpha-Ketoglutaric acid	HMDB0000208	494.6±214.9	135.0±5.01	30.8-398.2	150.5 (10.0-836.3)[10]
Citric acid	HMDB0000094	1833±1666	396.6±221	39.6-893.5	46-1400[a] [4]
Succinic acid	HMDB0000254	91.3±69.2	21.8±15.4	4.35-81.2	9-360[a] [4]
Fumaric acid	HMDB0000134	59.6±42.2	14.2±9.01	2.09-37.7	10.4 (2.8-53.7)[c] [10]
Pyruvic acid	HMDB0000243	163.5±96.3	36.9±15.1	19.6-89.1	21.2 (8.8-59.3)[c] [10]
Hippuric acid	HMDB0000714	261.5±285.7	64.1±63.2	1.51-213.0	49-150[a] [4]
Methylmalonic acid	HMDB0000202	12.3±17.0	2.79±3.60	0.42-19.7	10-20[a] [4]
Homovanillic acid	HMDB0000118	2.08±1.76	0.64±0.54	0.04-1.99	0-25[a] [11]
Indoleacetic acid	HMDB0000197	1.68±1.46	0.41±0.41	0.03-1.95	5.2 (0.1-31.0)[c] [10]
Dopamine	HMDB0000073	7.86±9.00	1.17±0.72	0.13-2.63	0.03-2.28*[a] [12]
Kynurenine	HMDB0000684	0.81±0.94	0.16±0.17	0.02-0.71	0.56 ± 0.91*[b] [5]
Butyric acid	HMDB0000039	1.05±0.12	0.31±0.19	0.06-0.86	3.2 (0.0-6.4)*[c] [3]
HPHPA	HMDB0002643	2.83±4.50	0.58±0.83	0.01-3.37	0.3-12.7*[a] [13]

Isobutyric acid	HMDB0001873	1.34±0.28	0.11±0.20	0.02-1.05	15.0 (12.0-27.0) *[c] [3]
Uric acid	HMDB0000289	6421±2585	1780±1057	218.8-4202	2000-4000*[a] [14]
Valerylcarnitine (C5)	HMDB0013128	0.26±0.27	0.06±0.05	0.02-0.29	0-3.0*[a] [15]

*Infant (0-1-year-old), [a] Data expressed as interquartile range, [b] Data expressed as mean ± SD , [c] Data expressed as median (range)

Supplementary Table 3: Creatinine urinary concentration in newborns

Metabolite	HMDB ID	Mean ± SD μM	2.5% - 97.5% Percentile μM	Literature values (μM)
**Creatinine**	HMDB0000562	4952.0±3381.0	1083.0-16760.0	2300.0 (980.0-10520.0)[3]

References for Supplementary Tables 2 and 3

1. Casado, M.; Sierra, C.; Batllori, M.; Artuch, R.; Ormazabal, A. A targeted metabolomic procedure for amino acid analysis in different biological specimens by ultra-high-performance liquid chromatography-tandem mass spectrometry. *Metabolomics* **2018**, *14*, 76, doi:10.1007/s11306-018-1374-4.
2. Neurology Catalog, A.A., Quantitative, Random, Urine. Available online: https://neurology.testcatalog.org/show/AAPD.
3. Geigy Scientific Tables, t.R.e., pp. 93. Edited by C. Lentner, West Cadwell, N.J.: Medical education Div., Ciba-Geigy Corp. Basel, Switzerland c1981-1992.
4. Embade, N.; Cannet, C.; Diercks, T.; Gil-Redondo, R.; Bruzzone, C.; Anso, S.; Echevarria, L.R.; Ayucar, M.M.M.; Collazos, L.; Lodoso, B., et al. NMR-based newborn urine screening for optimized detection of inherited errors of metabolism. *Sci Rep* **2019**, *9*, 13067, doi:10.1038/s41598-019-49685-x.
5. Shoemaker, J.D.; Elliott, W.H. Automated screening of urine samples for carbohydrates, organic and amino acids after treatment with urease. *J Chromatogr* **1991**, *562*, 125-138, doi:10.1016/0378-4347(91)80571-s.
6. Parvy, P.R.; Bardet, J.I.; Rabier, D.M.; Kamoun, P.P. Age-related reference values for free amino acids in first morning urine specimens. *Clin Chem* **1988**, *34*, 2092-2095.
7. Auray-Blais, C.; Maranda, B.; Lavoie, P. High-throughput tandem mass spectrometry multiplex analysis for newborn urinary screening of creatine synthesis and transport disorders, Triple H syndrome and OTC deficiency. *Clin Chim Acta* **2014**, *436*, 249-255, doi:10.1016/j.cca.2014.05.024.
8. Xing, X.; Liu, J.S.; Zhong, W. MetaGen: reference-free learning with multiple metagenomic samples. *Genome Biol* **2017**, *18*, 187, doi:10.1186/s13059-017-1323-y.
9. Aygen, S.; Durr, U.; Hegele, P.; Kunig, J.; Spraul, M.; Schafer, H.; Krings, D.; Cannet, C.; Fang, F.; Schutz, B., et al. NMR-Based Screening for Inborn Errors of Metabolism: Initial Results from a Study on Turkish Neonates. *JIMD Rep* **2014**, *16*, 101-111, doi:10.1007/8904_2014_326.
10. Guneral, F.; Bachmann, C. Age-related reference values for urinary organic acids in a healthy Turkish pediatric population. *Clin Chem* **1994**, *40*, 862-866.
11. Fitzgibbon, M.C.; Tormey, W.P. Paediatric reference ranges for urinary catecholamines/metabolites and their relevance in neuroblastoma diagnosis. *Ann Clin Biochem* **1994**, *31 (Pt 1)*, 1-11, doi:10.1177/000456329403100101.
12. Davidson, D.F.; Hammond, P.J.; Murphy, D.; Carachi, R. Age-related medical decision limits for urinary free (unconjugated) metadrenalines, catecholamines and metabolites in random urine specimens from children. *Ann Clin Biochem* **2011**, *48*, 358-366, doi:10.1258/acb.2011.011023.
13. Shaw, W. Increased urinary excretion of a 3-(3-hydroxyphenyl)-3-hydroxypropionic acid (HPHPA), an abnormal phenylalanine metabolite of Clostridia spp. in the gastrointestinal tract, in urine samples from patients with autism and schizophrenia. *Nutr Neurosci* **2010**, *13*, 135-143, doi:10.1179/147683010X12611460763968.
14. Aukett, A.; Bennett, M.J.; Hosking, G.P. Molybdenum co-factor deficiency: an easily missed inborn error of metabolism. *Dev Med Child Neurol* **1988**, *30*, 531-535, doi:10.1111/j.1469-8749.1988.tb04781.x.
15. Korman, S.H.; Andresen, B.S.; Zeharia, A.; Gutman, A.; Boneh, A.; Pitt, J.J. 2-ethylhydracrylic aciduria in short/branched-chain acyl-CoA dehydrogenase deficiency: application to diagnosis and implications for the R-pathway of isoleucine oxidation. *Clin Chem* **2005**, *51*, 610-617, doi:10.1373/clinchem.2004.043265.
