# Supplementary material for: The Urinary Metabolome of Healthy Newborns
Source: Metabolites. 2020 Apr 23;10(4):165. doi: 10.3390/metabo10040165 (PMC7240964; doi:10.3390/metabo10040165)
Supplement: Supplementary file 1 [file metabolites-10-00165-s001.zip › sp/Supplementary Table 4. Differences in gender (Amino acids and biogenic amines).pdf]

metabolites

Supplementary Table 4. Differences in gender (Amino acids and biogenic amines)

Metabolite	HMDB ID	Male n=31 (µM/mM creatinine)	Female n=17 (µM/mM creatinine)	P value	Test
Glycine	HMDB0000123	281.7(101.8-764.4)	374.4(180.9-879.5)	0.3577	Mann-Whitney test
Alanine	HMDB0000161	79.8±30.5	70.7±24.4	0.2945	T test
Serine	HMDB0000187	79.1±29.9	76.9±22.7	0.7913	T test
Histamine	HMDB0000870	0.02±0.01	0.02±0.01	0.2352	T test
Proline	HMDB0000162	23(9.49-82.4)	22.4(12.7-53.6)	0.4942	Mann-Whitney test
Valine	HMDB0000883	6.57(0.74-17.0)	5.07(1.87-18.9)	0.2057	Mann-Whitney test
Threonine	HMDB0000167	30.7(9.1-99.5)	25.4(15.2-69.4)	0.4545	Mann-Whitney test
Taurine	HMDB0000251	1042.0±591.5	1114.0±761.3	0.7176	T test
Putrescine	HMDB0001414	0.12(0.03-3.68)	0.16(0.05-1.14)	0.4545	Mann-Whitney test
4-Hydroxyproline	HMDB0000725	34.7(8.07-96.6)	28.4(17.0-78.8)	0.2472	Mann-Whitney test
Leucine	HMDB0000687	6.26±2.87	5.40±2.87	0.3251	T test
Isoleucine	HMDB0000172	2.93±1.42	2.73±1.52	0.6552	T test
Asparagine	HMDB0000168	2.68(0.36-10.6)	2.81(1.84-5.77)	0.8715	Mann-Whitney test
Aspartic acid	HMDB0000191	16.90±7.25	16.90±7.16	0.3696	T test
Glutamine	HMDB0000641	50.0(15.5-135.8)	50.8(32.4-129.3)	0.6084	Mann-Whitney test
Glutamic acid	HMDB0000148	11.5±7.0	14.3±10.1	0.2743	T test
Methionine	HMDB0000696	2.29±0.94	2.17±0.77	0.6549	T test
Dopamine	HMDB0000073	1.10±0.70	1.31±0.76	0.3918	T test
Histidine	HMDB0000177	60.3(18.7-193.6)	53.5(29.2-137.2)	0.8478	Mann-Whitney test
a-Aminoadipic acid	HMDB0000510	6.09±3.36	8.52±4.45	0.0444*	Mann-Whitney test
Phenylalanine	HMDB0000159	7.57±2.52	7.59±2.54	0.9783	T test
Methionine sulfoxide	HMDB0002005	1.67±0.69	1.48±0.80	0.3919	T test
Arginine	HMDB0000517	14.70±4.52	16.40±6.91	0.3216	T test
N2-Acetylornithine	HMDB0003357	0.41(0.12-3.50)	0.42(0.14-3.3)	0.9660	Mann-Whitney test
Serotonin	HMDB0000259	0.20±0.05	0.20±0.07	0.8491	T test
Tyrosine	HMDB0000158	5.96±2.98	5.82±3.26	0.8811	T test
DOPA	HMDB0000181	0.04(0.02-0.17)	0.04(0.01-0.08)	0.1763	Mann-Whitney test
ADMA	HMDB0001539	2.30±0.97	2.34±0.52	0.8596	T test
Symmetric dimethylarginine	HMDB0003334	8.86±2.16	10.90±3.86	0.0229*	T test
Tryptophan	HMDB0000929	3.44±0.99	3.22±0.97	0.4584	T test
Kynurenine	HMDB0000684	0.10(0.02-0.71)	0.13(0.02-0.68)	0.7408	Mann-Whitney test
Carnosine	HMDB0000033	2.67(0.45-36.20)	3.24(0.55-33.00)	0.8478	Mann-Whitney test
Ornithine	HMDB0000214	5.79(1.28-25.50)	4.72(2.03-10.50)	0.3356	Mann-Whitney test
Lysine	HMDB0000182	51.4±33.3	38.1±15.5	0.1648	Mann-Whitney test
Spermidine	HMDB0001257	0.04(0.01-0.08)	0.06(0.02-0.13)	0.0286*	Mann-Whitney test
Spermine	HMDB0001256	0.03(0.01-0.08)	0.05(0.01-0.13)	0.0027*	Mann-Whitney test
Sarcosine	HMDB0000271	2.28(0.51-4.79)	1.74(0.77-11.40)	0.6851	Mann-Whitney test
N1,N12-Diacetylspermine	HMDB0002172	1.03±0.64	1.02±0.51	0.9452	T test
Tyramine	HMDB0000306	0.97±0.40	0.91±0.39	0.7086	T test
Creatine	HMDB0000064	14.9(4.7-455.8)	13.4(4.6-104.0)	0.4044	Mann-Whitney test
Betaine	HMDB0000043	194.3±76.0	181.0±57.8	0.5356	T test
Choline	HMDB0000097	15.90(4.53-51.50)	16.10(3.95-36.50)	0.9694	Mann-Whitney test
Trimethylamine N-oxide	HMDB0000925	8.32(0.08-45.20)	11.40(1.32-30.80)	0.6043	Mann-Whitney test
1-Methylhistidine<br>3-Methylhistidine	HMDB0000001<br>HMDB0000479	47.1(17.3-146.8)	53.1(22.8-139.9)	0.1113	Mann-Whitney test

*Statistically significant (p<0.05)
