# Supplementary material for: The Urinary Metabolome of Healthy Newborns
Source: Metabolites. 2020 Apr 23;10(4):165. doi: 10.3390/metabo10040165 (PMC7240964; doi:10.3390/metabo10040165)
Supplement: Supplementary file 1 [file metabolites-10-00165-s001.zip › sp/Supplementary Table 5. Differences in gender (Organic acids and glucose).pdf]

metabolites

MDPI

Supplementary Table 5. Differences in gender (Organic acids and glucose)

Metabolite	HMDB ID	Male n=31 (μM/mM creatinine)	Female n=17 (μM/mM creatinine)	P value	Test
Lactic acid	HMDB0000190	100.0(51.0-797.5)	114.1(47.0-359.8)	0.6972	Mann-Whitney test
3-Hydroxybutyric acid	HMDB0000357	3.14(0.71-23.80)	4.02(0.78-12.40)	0.6818	Mann-Whitney test
alpha-Ketoglutaric acid	HMDB0000208	104.8(30.2-413.8)	90.8(45.6-331.1)	0.9151	Mann-Whitney test
Citric acid	HMDB0000094	382.2±208.3	422.9±246.8	0.5473	T test
Butyric acid	HMDB0000039	0.34±0.20	0.22±0.08	0.0312*	T test
HPHPA	HMDB0002643	0.18(0.01-3.37)	0.20(0.05-1.57)	0.6888	Mann-Whitney test
p-Hydroxyhippuric acid	HMDB0013678	7.48(4.17-20.0)	7.42(4.69-18.3)	0.8145	Mann-Whitney test
Succinic acid	HMDB0000254	16.8(3.97-85.2)	17.0(5.66-67.5)	0.8478	Mann-Whitney test
Fumaric acid	HMDB0000134	13.3±7.3	15.8±11.6	0.3594	T test
Pyruvic acid	HMDB0000243	34.2(19.3-93.0)	33.9(23.6-47.7)	0.5357	Mann-Whitney test
Isobutyric acid	HMDB0001873	0.06(0.02-1.07)	0.06(0.02-0.98)	0.6387	Mann-Whitney test
Hippuric acid	HMDB0000714	60.0(3.2-207.3)	28.7(1.3-213.6)	0.4042	Mann-Whitney test
Methylmalonic acid	HMDB0000202	1.89(0.42-22.50)	1.28(0.52-6.64)	0.2300	Mann-Whitney test
Homovanillic acid	HMDB0000118	0.43(0.04-2.00)	0.50(0.06-1.78)	0.8109	Mann-Whitney test
Indoleacetic acid	HMDB0000197	0.28(0.02-1.98)	0.22(0.06-1.18)	0.6695	Mann-Whitney test
Uric acid	HMDB0000289	2024.0±1084.0	1334.0±865.8	0.0288*	T test
Glucose	HMDB0000122	421.0± 219.7	507.9±275.0	0.2409	Mann-Whitney test

*Statistically significant ($p < 0.05$)
