# Supplementary material for: The Urinary Metabolome of Healthy Newborns
Source: Metabolites. 2020 Apr 23;10(4):165. doi: 10.3390/metabo10040165 (PMC7240964; doi:10.3390/metabo10040165)
Supplement: Supplementary file 1 [file metabolites-10-00165-s001.zip › sp/Supplementary Table 6. Differences in gender (Glycerophospholipids).pdf]

metabolites MDPI

1 **Supplementary Table 6.** Differences in gender (Glycerophospholipids)

Metabolite	HMDB ID	Male n=31 (μM/mM creatinine)	Female n=17 (μM/mM creatinine)	p Value	Test
LysoPC a C14:0	HMDB0010379	0.006±0.003	0.0046±0.0025	0.1039	T test
LysoPC a C16:1	HMDB0010383	0.0020(0.0001-0.0069)	0.0027(0.0008-0.0150)	0.2217	Mann-Whitney test
LysoPC a C16:0	HMDB0010382	0.03(0.01-0.11)	0.03(0.01-0.18)	0.6695	Mann-Whitney test
LysoPC a C17:0	HMDB0012108	0.002(0.001-0.007)	0.006(0.002-0.009)	0.0001*[a]	Mann-Whitney test
LysoPC a C18:2	HMDB0010386	0.005(0.001-0.031)	0.007(0.001-0.029)	0.2217	Mann-Whitney test
LysoPC a C18:1	HMDB0002815	0.007(0.001-0.018)	0.016(0.004-0.057)	0.008*[a]	Mann-Whitney test
LysoPC a C18:0	HMDB0010384	0.0094 (0.0006-0.0340)	0.016(0.005-0.069)	0.0887	Mann-Whitney test
LysoPC a C20:4	HMDB0010395	0.0031(0.0001-0.0310)	0.0035(0.0001-0.0240)	0.9830	Mann-Whitney test
LysoPC a C20:3	HMDB0010394	0.004±0.003	0.003±0.001	0.3437	T test
LysoPC a C24:0	HMDB0010405	0.02±0.01	0.02±0.01	0.6397	T test
LysoPC a C26:1	HMDB0029220	0.0012 (0.0001-0.0130)	0.0014(0.0001-0.0037)	0.4830	Mann-Whitney test
LysoPC a C26:0	HMDB0029205	0.0021(0.0001-0.0100)	0.0032(0.0008-0.0340)	0.1203	Mann-Whitney test
LysoPC a C28:1	HMDB0029221	0.0016(0.0001-0.0110)	0.0014(0.0001-0.0110)	0.8310	Mann-Whitney test
LysoPC a C28:0	HMDB0029206	0.01±0.01	0.01±0.01	0.7599	T test
PC aa C32:2	HMDB0007874	0.0044(0.0005-0.0190)	0.0057(0.0018-0.0320)	0.3924	Mann-Whitney test
PC ae C36:0	HMDB0013406	0.0029±0.0022	0.0041±0.0023	0.0725	T test
PC aa C36:6	HMDB0008206	0.0013±0.0009	0.0019±0.0014	0.0544	T test
PC aa C36:0	HMDB0007886	0.006 (0.001-0.024)	0.011(0.004-0.030)	0.0259*[a]	Mann-Whitney test
PC aa C38:6	HMDB0008116	0.008 (0.002-0.094)	0.012(0.004-0.054)	0.1980	Mann-Whitney test
PC aa C38:0	HMDB0007893	0.02±0.01	0.02±0.01	0.3590	T test
PC ae C40:6	HMDB0013422	0.004(0.001-0.014)	0.004 (0.002-0.023)	0.3924	Mann-Whitney test
PC aa C40:6	HMDB0008057	0.0057(0.0008-0.0170)	0.0083(0.0021-0.0880)	0.2057	Mann-Whitney test
PC aa C40:2	HMDB0008276	0.0006(0.0001-0.0033)	0.0016(0.0004-0.0590)	0.0318*[a]	Mann-Whitney test
PC aa C40:1	HMDB0007993	0.0034(0.0014-0.0110)	0.0058(0.0023-0.0130)	0.0300*[a]	Mann-Whitney test

2 *Statistically significant ($p < 0.05$)

3 [a] Significative after multiple correction testing (False Discovery Rate (FDR) < 0.05))
