# Supplementary material for: The Urinary Metabolome of Healthy Newborns
Source: Metabolites. 2020 Apr 23;10(4):165. doi: 10.3390/metabo10040165 (PMC7240964; doi:10.3390/metabo10040165)
Supplement: Supplementary file 1 [file metabolites-10-00165-s001.zip › sp/Supplementary Table 7. Differences in gender (Sphingomyelins).pdf]

metabolites

Supplementary Table 7. Differences in gender (Sphingomyelins)

Metabolite	HMDB ID	Male n=31 (μM/mM creatinine)	Female n=17 (μM/mM creatinine)	p Value	Test
SM(OH) C14:1	HMDB0013462	0.014(0.004-0.067)	0.017(0.006-0.290)	0.1268	Mann-Whitney test
SM C16:1	HMDB0013464	0.013(0.004-0.099)	0.019(0.005-0.110)	0.2843	Mann-Whitney test
SM C16:0	HMDB0010168	0.19(0.01-0.69)	0.44(0.09-1.11)	0.0234*	Mann-Whitney test
SM(OH) C16:1	HMDB0013463	0.0062(0.0008-0.0250)	0.012(0.001-0.049)	0.0264*[a]	Mann-Whitney test
SM C18:1	HMDB0012101	0.008(0.003-0.060)	0.011(0.004-0.057)	0.2472	Mann-Whitney test
SM C18:0	HMDB0012087	0.03 (0.01-0.25)	0.07(0.02-0.49)	0.1380	Mann-Whitney test
SM C20:2	HMDB0013465	0.0009(0.0001-0.0041)	0.0010(0.0002-0.0045)	0.5464	Mann-Whitney test
SM(OH) C22:2	HMDB0013467	0.0032(0.0001-0.0280)	0.0081(0.0015-0.0510)	0.0929	Mann-Whitney test
SM(OH) C22:1	HMDB0013466	0.033±0.022	0.041±0.036	0.3724	T test
SM(OH) C24:1	HMDB0013469	0.005±0.003	0.01±0.01	0.0005*[a]	T test

*Statistically significant ($p < 0.05$)

[a] Significant after multiple correction testing (False Discovery Rate (FDR < 0.05))
