# Supplementary material for: The Urinary Metabolome of Healthy Newborns
Source: Metabolites. 2020 Apr 23;10(4):165. doi: 10.3390/metabo10040165 (PMC7240964; doi:10.3390/metabo10040165)
Supplement: Supplementary file 1 [file metabolites-10-00165-s001.zip › sp/Supplementary Table 8. Differences in gender (acylcarnitines).pdf]

1 **Supplementary Table 8.** Differences in gender (acylcarnitines)

Metabolite	HMDB ID	Male n=31 (µM/mM creatinine)	Female n=17 (µM/mM creatinine)	p Value	Test
Carnitine (C0)	HMDB0000062	1.80(0.78-5.85)	1.63(0.83-4.31)	0.2385	Mann-Whitney test
L-Acetylcarnitine (C2)	HMDB0000201	0.92±0.39	0.85±0.38	0.5282	T test
Propenoylcarnitine (C3:1)	HMDB0013124	0.017(0.004-0.064)	0.013(0.006-0.046)	0.4675	Mann-Whitney test
Propionylcarnitine (C3)	HMDB0000824	0.034±0.017	0.033±0.016	0.8246	T test
Butenylcarnitine (C4:1)	HMDB0013126	0.024±0.009	0.021±0.007	0.4136	T test
Butyrylcarnitine (C4)	HMDB0002013	0.13±0.07	0.11±0.06	0.2853	T test
Hydroxypropionylcarnitine (C3OH)	HMDB0013125	0.03±0.01	0.02±0.01	0.0798	T test
Tiglylcarnitine (C5:1)	HMDB0002366	0.07±0.02	0.08±0.03	0.4633	T test
Valerylcarnitine (C5)	HMDB0013128	0.05(0.02-0.29)	0.04 (0.02-0.25)	0.5079	Mann-Whitney test
Hydroxybutyrylcarnitine (C4OH)	HMDB0002095	0.04±0.02	0.04±0.02	0.4431	T test
Hexenoylcarnitine (C6:1)	HMDB0013161	0.014±0.004	0.013±0.003	0.4372	T test
Hexanoylcarnitine (C6)	HMDB0000756	0.028±0.008	0.026±0.007	0.3726	T test
Hydroxyvalerylcarnitine (C5OH)	HMDB0013132	0.08(0.04-0.33)	0.06 (0.05-0.13)	0.2057	Mann-Whitney test
Glutaconylcarnitine (C5:1DC)	HMDB0013129	0.017±0.007	0.014±0.006	0.2506	T test
Glutarylcarnitine (C5DC)	HMDB0013130	0.05±0.02	0.05±0.02	0.9269	T test
Octanoylcarnitine (C8)	HMDB0000791	0.04 (0.02-0.10)	0.05 (0.02-0.37)	0.9320	Mann-Whitney test
Methylglutarylcarnitine (C5MDC)	HMDB0000552	0.06(0.03-0.13)	0.05 (0.03-0.13)	0.6235	Mann-Whitney test
Nonaylcarnitine (C9)	HMDB0013288	0.08 (0.04-0.20)	0.10(0.03-0.15)	0.9320	Mann-Whitney test
Pimelylcarnitine (C7DC)	HMDB0013328	0.05±0.02	0.04±0.02	0.5441	T test
Decadienylcarnitine (C10:2)	HMDB0013325	0.03 (0.02-0.07)	0.03(0.01-0.05)	0.1833	Mann-Whitney test
Decenoylcarnitine (C10:1)	HMDB0013205	0.09±0.04	0.08±0.04	0.3205	T test
Decanoylcarnitine (C10)	HMDB0000651	0.06 (0.04-0.09)	0.06(0.04-0.14)	0.6540	Mann-Whitney test
Dodecenoylcarnitine (C12:1)	HMDB0013326	0.06±0.02	0.06±0.02	0.9001	T test
Dodecanoylcarnitine (C12)	HMDB0002250	0.07 (0.04-0.28)	0.07(0.03-0.14)	0.8813	Mann-Whitney test
Tetradecadienylcarnitine (C14:2)	HMDB0013331	0.011±0.003	0.010±0.003	0.9267	T test
Tetradecenoylcarnitine (C14:1)	HMDB0013329	0.009 (0.004-0.110)	0.010 (0.006-0.054)	0.2136	Mann-Whitney test
Tetradecanoylcarnitine (C14)	HMDB0005066	0.02 (0.01-0.07)	0.02(0.01-0.09)	0.6695	Mann-Whitney test
Dodecanedioylcarnitine (C12DC)	HMDB0013327	0.04 (0.02-0.15)	0.04(0.02-0.33)	0.7168	Mann-Whitney test
Hydroxytetradecadienylcarnitine (C14:2OH)	HMDB0013332	0.007(0.004-0.012)	0.006(0.003-0.015)	0.1268	Mann-Whitney test
Hydroxytetradecenoylcarnitine (C14:1OH)	HMDB0013330	0.007±0.001	0.008±0.003	0.2756	T test
Hexadecadienylcarnitine (C16:2)	HMDB0013334	0.005±0.002	0.004±0.001	0.1857	T test
Hexadecenoylcarnitine (C16:1)	HMDB0006317	0.012±0.005	0.011±0.006	0.3055	T test
Hexadecanoylcarnitine (C16)	HMDB0000222	0.011(0.006-0.028)	0.011(0.006-0.071)	0.1833	Mann-Whitney test
Hydroxyhexadecadienylcarnitine (C16:2OH)	HMDB0013335	0.004(0.002-0.007)	0.003 (0.002-0.007)	0.3924	Mann-Whitney test
Hydroxyhexadecenoylcarnitine (C16:1OH)	HMDB0013333	0.008 (0.004-0.015)	0.008(0.004-0.027)	0.2562	Mann-Whitney test
Hydroxyhexadecanoylcarnitine (C16OH)	HMDB0061642	0.008±0.003	0.008±0.003	0.8295	T test

Octadecadienylcarnitine (C18:2)	HMDB0006469	0.003(0.001-0.007)	0.002(0.001-0.057)	0.0178*	Mann-Whitney test
Octadecenoylcarnitine (C18:1)	HMDB0006464	0.004 (0.002-0.010)	0.004 (0.003-0.033)	0.8645	Mann-Whitney test
Octadecanoylcarnitine (C18)	HMDB0000848	0.005 (0.002-0.023)	0.007 (0.003-0.021)	0.0945	Mann-Whitney test
Hydroxyoctadecenoylcarnitine (C18:1OH)	HMDB0013339	0.004 (0.002-0.008)	0.004 (0.002-0.011)	0.8982	Mann-Whitney test

*Statistically significant ($p < 0.05$)
