# Supplementary material for: The Urinary Metabolome of Healthy Newborns
Source: Metabolites. 2020 Apr 23;10(4):165. doi: 10.3390/metabo10040165 (PMC7240964; doi:10.3390/metabo10040165)
Supplement: Supplementary file 1 [file metabolites-10-00165-s001.zip › sp/Supplementary Table 9. List of metabolites experimentally measured in the present work that are currently used as primary or secondary markers .pdf]

metabolites

MDPI

Supplementary Table 9. List of metabolites experimentally measured in the present work that are currently used as primary or secondary markers for Inborn errors of metabolism

URINARY MARKER	DISEASE	ABNORMAL CONCENTRATION (μM/mM creatinine)	REFERENCE
Glycine	Non-ketotic hyperglycinaemia	4753.0-6114.0	[1]
	Isovaleric acidemia	500.0 – 5000.0	[2]
	Propionic acidemia	2000.0-2500.0	[2]
	Ornithine transcarbamilase deficiency	2000.0-5000.0	[2]
	Arginosuccinic aciduria	2000.0-5000.0	[2]
	Transient immaturity of transporters	2000.0-6000.0	[2]
	MMA Cbl A deficiency	2000.0-5000.0	[2]
Alanine	Hartnup disease	400.0-1400.0	[2]
	Piruvate carboxylase deficiency	700.0-1400.0	[2]
	Ethylmalonic encephalopathy	70.0-1400.0	[2]
	Amish lethal microcephaly	427.6-681.4	[3]
Valine	Maple syrup urine disease (MSUD)	20.0-50.0	[2]
	Hartnup disease	40.0-500.0	[2]
Leucine	Hartnup disease	50.0-200.0	[2]
	Mapple Sirup Urine disease	80.0-240.0	[2]
Isoleucine	Hartnup disease	50.0-500.0	[2]
	Mapple Sirup Urine disease	50.0-500.0	[2]
Glutamine	Hartnup disease	300.0-3000.0	[2]
	Fumaric aciduria	154.0-512.0	[4]
	Glutamine deficiency, congenital	0.00-8.00	[5]

Phenylalanine	Phenylketonuria	> 414.76	[6]
Tyrosine	Tyrosinemia (Tyr)	> 150.0	[2]
	Methylmalonic aciduria	150.0-15500.0	[2]
	Hartnup disease	50.0-500.0	[2]
Tryptophan	Hartnup disease	80.0-240.0	[2]
Ornithine	Hyperdibasic aminoaciduria I	8.90-14.0	[7]
	Lysinuric protein intolerance	16.1-51.00	[8]
	Cystinuria	350.0 (200.0-500.0)	http://www.metagene.de
Sarcosine	Sarcosinemia	100.0-5000.0	[2]
	Glutaric aciduria type II	25.0-100.0	[2]
Tyramine	Dihydropyriminidase deficiency	> 400.0	[2]
Betaine	Dimethylglycine dehydrogenase deficiency	1200.0-5000.0	[2]
	Homocistinuria	1200.0-5000.0	[2]
4-Hydroxyproline	Iminoglycinuria	0.00-250.0	[9]
	Hydroxyprolinemia	> 2960.0	[10]
Lactic acid	Primary lactic acidemia	500.0-3000.0	[2]
	3-Methyl-glutaconic aciduria type IV	500.0-1000.0	[2]
	Biotinidase deficiency	500.0-75000.0	[2]
	Pyruvate decarboxylase deficiency	500.0-3000.0	[2]
	Ethylmalonic encephalopathy	400.0-3000.0	[2]
beta-Hydroxybutyric acid	Propionic acidemia	100.0-5000.0	[2]
	Long Chain 3-HydroxyacylCoA Dehydrogenase deficiency	100.0-5000.0	[2]

	Maple Sirup Urine disease	200.0-1000.0	[2]
	Pyruvate Carboxylase eficiency	200.0-600.0	[2]
	b-Ketothiolase deficiency	50.0 – 200.0	[2]
	Fructose-1,6- biphosphatase deficiency	4.00– 48.0	[2]
	Glutaric aciduria type I	200.0 – 800.0	[2]
	Isovaleric acidemia	200.0 – 1000.0	[2]
Citric acid	3-Methylglutaconic aciduria	500.0-25000.0	[2]
Succinic acid	2-Ketoadipic acidemia	400.0-1200.0	[2]
	3-Methyl-glutaconic aciduria type II	400.0-1200.0	[2]
	Ethylmalonic encephalopathy	400.0-1200.0	[2]
	D-2-Hydroxyglutaric aciduria	> 86.16	[11]
	Pyruvate carboxylase deficiency	400.0-1200.0	[2]
	Fumaric aciduria	400.0-1200.0	[2]
Fumaric acid	2-Ketoadipic acidemia	100.0-1000.0	[2]
	Fumaric aciduria	3000.0-4000.0	[2]
	Ethylmalonic encephalopathy	> 100.0	[2]
Pyruvic acid	3-Methyl-glutaconic aciduria	50.0-200.0	[2]
Hippuric acid	Argininemia	200.0-5000.0	[2]
	Argininosuccinic aciduria	1000.0-15000.0	[2]
	Citrullinemia	200.0-50000.0	[2]
	Transient infantile liver failure	1000.0-15000.0	[2]
	Ornithine transcarbamilase deficiency	300.0-10000.0	[2]

Methylmalonic acid	Isovaleric aciduria	50.0-500.0	[2]

References for Supplementary Table 9

1. Brandt, N.J.; Brandt, S.; Rasmussen, K.; Schnoheyder, F. Letter: Hyperglycericacidaemia with hyperglycinaemia: a new inborn error of metabolism. *Br Med J* **1974**, *4*, 344, doi:10.1136/bmj.4.5940.344-a.
2. Embade, N.; Cannet, C.; Diercks, T.; Gil-Redondo, R.; Bruzzone, C.; Anso, S.; Echevarria, L.R.; Ayucar, M.M.M.; Collazos, L.; Lodoso, B., et al. NMR-based newborn urine screening for optimized detection of inherited errors of metabolism. *Sci Rep* **2019**, *9*, 13067, doi:10.1038/s41598-019-49685-x.
3. Kelley, R.I.; Robinson, D.; Puffenberger, E.G.; Strauss, K.A.; Morton, D.H. Amish lethal microcephaly: a new metabolic disorder with severe congenital microcephaly and 2-ketoglutaric aciduria. *Am J Med Genet* **2002**, *112*, 318-326, doi:10.1002/ajmg.10529.
4. Allegri, G.; Fernandes, M.J.; Scalco, F.B.; Correia, P.; Simoni, R.E.; Llerena, J.C., Jr.; de Oliveira, M.L. Fumaric aciduria: an overview and the first Brazilian case report. *J Inherit Metab Dis* **2010**, *33*, 411-419, doi:10.1007/s10545-010-9134-2.
5. Haberle, J.; Gorg, B.; Rutsch, F.; Schmidt, E.; Toutain, A.; Benoist, J.F.; Gelot, A.; Suc, A.L.; Hohne, W.; Schliess, F., et al. Congenital glutamine deficiency with glutamine synthetase mutations. *N Engl J Med* **2005**, *353*, 1926-1933, doi:10.1056/NEJMoa050456.
6. Swarna, M.; Jyothy, A.; Usha Rani, P.; Reddy, P.P. Amino acid disorders in mental retardation: a two-decade study from Andhra Pradesh. *Biochem Genet* **2004**, *42*, 85-98, doi:10.1023/b:bigi.0000020464.05335.79.
7. Whelan, D.T.; Scriver, C.R. Hyperdibasicaminoaciduria: an inherited disorder of amino acid transport. *Pediatr Res* **1968**, *2*, 525-534, doi:10.1203/00006450-196811000-00011.
8. Habib, A.; Azize, N.A.; Yakob, Y.; Md Yunus, Z.; Wee, T.K. Biochemical and molecular characteristics of Malaysian patients with lysinuric protein intolerance. *Malays J Pathol* **2016**, *38*, 305-310.
9. Broer, S.; Bailey, C.G.; Kowalczuk, S.; Ng, C.; Vanslambrouck, J.M.; Rodgers, H.; Auray-Blais, C.; Cavanaugh, J.A.; Broer, A.; Rasko, J.E. Iminoglycinuria and hyperglycinuria are discrete human phenotypes resulting from complex mutations in proline and glycine transporters. *J Clin Invest* **2008**, *118*, 3881-3892, doi:10.1172/JCI36625.
10. la Marca, G.; Malvagia, S.; Pasquini, E.; Donati, M.A.; Gasperini, S.; Procopio, E.; Zammarchi, E. Hyperhydroxyprolinaemia: a new case diagnosed during neonatal screening with tandem mass spectrometry. *Rapid Commun Mass Spectrom* **2005**, *19*, 863-864, doi:10.1002/rcm.1861.
11. Chalmers, R.A.; Lawson, A.M.; Watts, R.W.; Tavill, A.S.; Kamerling, J.P.; Hey, E.; Ogilvie, D. D-2-hydroxyglutaric aciduria: case report and biochemical studies. *J Inherit Metab Dis* **1980**, *3*, 11-15, doi:10.1007/bf02312516.
